# Failure to Comprehend Determinism or Failure to Measure Comprehension? Methodological Issues in Experimental Philosophy of Free Will

**DOI:** 10.1007/s10670-024-00844-1

**Published:** 2024-08-20

**Authors:** Florian Cova, Tristan Martinez

**Affiliations:** 1 Swiss Center for Affective Sciences, University of Geneva, Geneva, Switzerland; 2 Département de Philosophie, Université de Genève, 5, rue de Candolle, 1211 Geneva 4, Switzerland; 3 Philosophy Department, University of Geneva, Geneva, Switzerland

## Abstract

In the past 20 years, experimental philosophers have been investigating folk intuitions about the compatibility of determinism with free will and moral responsibility using vignettes depicting agents in deterministic universes. However, recent research suggests that participants massively fail to understand these vignettes. Moreover, it has also been proposed that these comprehension errors might even be systematic and thus unavoidable, threatening the project of probing folk intuitions about free will and determinism through vignettes. Through five studies, we suggest that past studies have overestimated participants’ failure to understand vignettes depicting deterministic universes, mainly by relying on unreliable samples and by using non-validated measures. We also show that using new vignettes that take more time to better describe what determinism is substantially reduces comprehension errors and their impact on participants’ intuitions. We conclude that, at least in the current state of the art, skepticism about the possibility to probe folk intuitions about the compatibility of determinism with free will and moral responsibility through the use of vignettes is premature.

**Supplementary Information:**

The online version contains supplementary material available at 10.1007/s10670-024-00844-1.

## Introduction

### Methodological Issues in Experimental Philosophy of Free Will

In the past 20 years, experimental philosophers have been investigating laypeople’s intuitions about free will and moral responsibility (Feltz, 2017). Most of this research has focused on whether laypeople are “natural compatibilists” or “natural incompatibilists” (Feltz et al., 2009), using vignettes describing deterministic universes and asking participants whether agents living in these universes can act of their own free will and be morally responsible for their actions (see Cova, 2023; Inarimori et al., 2024a for surveys).

However, this method soon ran into some difficulties. While the results of first studies suggested that most people were “natural compatibilists” (Nahmias et al., 2005, 2006), later studies suggested that participants’ answers depended on the content on the vignettes: abstract vignettes elicited more incompatibilist answers compared to concrete ones (Nichols & Knobe, 2007), while vignettes focusing on psychological determinism elicited more compatibilist intuitions than vignettes focusing on neuroscientific determinism (Nahmias et al., 2007). Searching to explain these conflicting results, Murray and Nahmias (2014) soon found out that a non-negligible proportion of participants presented with such vignettes tended to interpret certain deterministic vignettes as implying *bypassing* (i.e. the claim that agents’ mental states play no role in the production of their decisions and actions). This meant that many seemingly ‘incompatibilist’ answers in fact expressed the (rather trivial) intuition that one cannot act of one’s own free will when one’s decisions and actions have no connection to one’s mental states, and that it was necessary to control for participants’ comprehension errors to obtain a reliable estimate of the proportion of “natural compatibilists” and “natural incompatibilists” among participants.

But if certain seemingly ‘incompatibilist’ answers do not reflect a genuine commitment to incompatibilism but a failure to comprehend what experiments intended to convey, could it also be that certain seemingly ‘compatibilist’ answers do not reflect a genuine commitment to compatibilism either? This is what has been suggested by several researchers. For example, Mandelbaum and Ripley (2012) proposed that participants attributed moral responsibility as soon as they perceived that an agent had broken a norm, and Feltz and Millan (2015) proposed that participants attributed free will “no matter what”. However, both accounts were made implausible by the fact that it is easy to design cases in which agents break a moral norm but are not considered morally responsible for it (Andow & Cova, 2016). A more promising version of this idea is the claim, put forward by Nadelhoffer and colleagues (2020), according to which participants often fail to abide by the content of the vignette and attribute to agents the power to escape determinism (i.e. the unconditional ability to do otherwise) even when the vignette specifies the contrary. Indeed, using two classic vignettes in the experimental philosophy literature (the *Supercomputer* and *Rollback* vignettes), they found that many participants still attributed to agents in this vignette the unconditional ability to do otherwise. For example, in the *Supercomputer* case, they asked participants to rate on a scale from 0 to 100 what were the chances that the agent would not behave as the computer had predicted. More than half of participants gave an answer superior to 0, suggesting that they still attributed to the agents some power of escaping determinism.^1^ For reasons that will become clearer later, they call this sort of error *intrusion* errors.

The presence of errors such as *bypassing* and *intrusion* errors poses a problem for the interpretation of experimental philosophy studies using vignettes describing deterministic universes: indeed, to interpret participants’ answers as expressing a commitment to “natural compatibilism” or “natural incompatibilism”, we first need to make sure that their intuitions are adequately sensitive to the relevant features of these vignettes. This is what Nadelhoffer and colleagues (2020) call the tracking problem.

But how serious is the tracking problem? What proportion of participants actually fail to understand experimental philosophy vignettes in the relevant way? This is the question that two recent research papers have sought to address. In the first, Nadelhoffer and colleagues (2023) begin by identifying three possible types of comprehension errors: *Intrusion, Epiphenomenalism,* and *Fatalism*. *Intrusion*, as already mentioned, involves participants introducing indeterministic assumptions into their understanding of supposedly deterministic scenarios. Nadelhoffer and colleagues measure such comprehension errors through items such as “In Universe A, what people decide to do could have been different even if everything leading up to the decision had been exactly the same”. *Epiphenomenalism* is similar to what Murray and Nahmias (2014) called ‘Bypassing’: it is the claim that, in a deterministic system beliefs, desires, and other intentional mental states are causally inert. It is measured through items such as “In Universe A, John would have decided to have French Fries no matter what he wanted or believed”. Finally, *Fatalism* is the idea that whatever happens had to happen no matter what, even if prior events had been different. This is different from determinism, which claims that whatever happens in a deterministic universe had to happen *given the past states of this universe and the laws of nature*. This confusion is measured through items such as “In Universe A, John would have ended up having French Fries no matter what he tried to do”.

Through two studies, Nadelhoffer and colleagues (2023) observed that most participants failed at least one of these comprehension tasks. In their first study, they found that 57% of participants failed the Intrusion task, 91% failed the Epiphenomenalism task, and 90% failed the Fatalism task. In their second study, 71% failed the Intrusion task, 43% failed the Epiphenomenalism task, and 67% failed the Fatalism task. Pursuing Nadelhoffer and colleagues (2023)’s work, Murray and colleagues investigated the frequency with which participants made these three types of errors, by comparing their answers to seven different vignettes drawn from the experimental philosophy literature. They found that less than 3% of their participants made no mistake.

These extremely high rates of error lead Nadelhoffer and colleagues (2023) and Murray and colleagues (forthcoming) to conclude that we cannot trust the results of past studies on people’s intuitions about free will and determinism, as participants’ answers are likely to be distorted by these comprehension errors. As Nadelhoffer and colleagues (2023:2517) put it: “there is likely enough confusion concerning determinism baked into the existing evidence on folk intuitions about free will that these intuitions can’t reliably be used to shed light on the free will debate”.

### Are Comprehension Errors Avoidable or Systematic?

One possible reaction to these results is to think that the difficulties identified by recent research on participants’ failure to comprehend vignettes can be overcome by designing *better* materials—materials that will not have participants fall into this kind of error. Let’s call this the *optimistic* response.

Against this *optimistic* response, one could defend a *pessimistic* conclusion, according to which it is simply impossible to design materials that will allow researchers to prevent massive comprehension errors and probe genuine intuitions about free will and determinism. Murray and colleagues (forthcoming) sketch such a view, without necessarily endorsing it, under the name of “experimental nihilism”. Here is how they formulate it:

no matter how well-designed some vignette is, participants are not able to respond to the target content because they lack the core concepts implicated in such content.

But are there reasons to think experimental nihilism might be true? Actually, there are. One major reason in favor of this view is the idea that at least some of the errors participants fall into are not *accidents* but rather *systematic errors*, so that there would be no viable way of preventing them.

Let’s take for example participants who think that the deterministic universes described in vignettes also entail bypassing. At first, we might think that this is a mistake that should be easy to correct: after all, there is no *logical* connection between determinism and bypassing. But there could be a *psychological* one. For example, it could be that people’s concepts of choice and decision are such that they are incompatible with determinism, so that it is impossible (or at least very hard) for people to imagine that determined agents could actually make choices and take decisions based on their reasons and not be bypassed.

This at least what Rose and Nichols (2013) have suggested: according to them, it is not because people make bypassing errors that they do not attribute free will and moral responsibility, but because they deny free will that they perceive the agent as bypassed. Based on the results of several studies showing that participants have trouble seeing determined agents as able to make choices, but have no trouble considering them able to perform other mental actions such as adding numbers, they argue that “the idea that free choices aren’t determined is not just a peripheral fact about free choice–it is at the core of the idea of free choice” (2013:618). Similarly, based on a series of four studies, Bear and Knobe (2016) argued that people tend to see determinism itself as being incompatible with active behaviors, such as taking a decision or resisting one’s urges. Thus, it could be that participants’ concepts of choices and decisions are such that they are fundamentally at odds with determinism. If this is the case, then it might prove impossible to have most participants accept both that an agent is determined and is making choices and decisions.

The theory behind the *Intrusion* measures is actually very similar. This idea finds its source in research by Rose and colleagues (2017) on the way people deal with the possibility of predicting agents’ decisions and behavior from their neural activity. Through six studies, Rose and colleagues showed that participants failed to comply with vignettes describing possible worlds in which neuroscientific prediction is possible, as they “imported” indeterministic assumptions in their interpretation of the vignette (e.g. they would claim that someone with the exact same brain activity could have still acted otherwise). One might think that these comprehension errors are specific to neuroscientific scenarios, as it has been shown that people are intuitive dualists (Chudek et al., 2018), and that they therefore have trouble considering that genuine decisions can be the product of physical, mechanistic processes (Nahmias et al., 2007). However, Rose and colleagues rather surmise that this “intrusion” of metaphysical assumptions in participants’ interpretation of the vignettes is rather due to an indeterministic conception of agency.

*Intrusion* measures are thus supposed to measure the extent to which participants’ intuitive conception of human agency *intrudes* in their interpretation and comprehension of vignettes describing deterministic universes. The hypothesis is that, because people’s conceptions of agency is fundamentally indeterministic^2^ they have trouble keeping in mind that the agent described in this type of vignettes is determined, and end up attributing them abilities incompatible with determinism, such as the unconditional ability to do otherwise.

These different suggestions can thus be summarized in the following way: because people’s conception of human agency is fundamentally indeterministic, it might prove impossible to get rid of certain of the comprehension errors we have mentioned. Either participants keep in mind that the universe is deterministic, but then they perceive this universe as implying *bypassing*. Or they refrain from interpreting them as implying bypassing, but then their intuitive conception of agency *intrudes*, and they end up attributing indeterministic abilities to the agents. One way or the other, the vignette is not properly understood.

### Failure to Comprehend or Failure to Measure Comprehension?

Our review of the literature thus suggests three claims, that we can summarize as follows (from the least to the most controversial):

1. Experimenters should pay attention to comprehension errors when investigating folk intuitions about free will and determinism, as intuitions tainted by comprehension errors are not informative.
2. The vignettes experimental philosophers have been using so far elicit massive comprehension errors, meaning that no useful conclusions can be drawn from studies using them.
3. Future experiments will be unlikely to yield more useful results, as comprehension errors are systematic and thus unavoidable.

We completely agree with statements (1). Rather, in this paper, we dispute claims (2) and (3) to defend what we take as a more optimistic conclusion regarding the prospect of experimentally investigating folk intuitions about free will, moral responsibility and determinism.

Regarding claim (2), we think that the recent studies we have surveyed might have overestimated the rate of relevant comprehension errors elicited by classic experimental philosophy vignettes, particularly when it comes to the rate of *Intrusion* errors. This is important, because, even if we could not get rid of *Bypassing* and *Fatalism* errors, getting rid of (most) *Intrusion* errors would be enough to reach a lower-bound estimate of the rate of “natural compatibilists” in a population (a theoretically informative results in itself—for example, reaching a lower-bound estimate of 60% natural compatibilists would already have important theoretical implications). More generally, being more optimistic about classic vignettes would justify being more optimistic about future studies and would provide additional reasons against (3), as it would mean that we can build on past vignettes to improve them.

According to us, there are at least two reasons to think that recent estimates might have overestimated the rate of *Intrusion* errors in past studies:

(i) The first is that the *m*e*asures* used in these studies might not capture what they are supposed to capture. Indeed, most estimates of the rate of comprehension errors rely on the assumption that while participants are unable to properly understand the vignettes, they are sufficiently able to understand what their questions mean. But this is actually a substantial empirical claim, for which they provide little evidence. Given recent worries about the lack of “calibration” (i.e. validation) of newly introduced measures in experimental philosophy (see Cova, forthcoming), one might think that measures, rather than vignettes, might be at fault.

Indeed, besides this general methodological point, there are positive reasons to doubt the validity of *Intrusion* and *Fatalism* measures in particular. The first comes from the very studies we have already mentioned. For example, in Nadelhoffer and colleagues (2023)’ first study, they found that 57% of participants failed the Intrusion task, 91% failed the Epiphenomenalism task, and 90% failed the Fatalism task. But, assuming that participants interpret their questions in the intended way, that simply *should not be possible*. Indeed, Fatalism precludes Intrusion (if things *had to happen no matter what*, then it is simply false that the agent *could have done otherwise*, both in a conditional and unconditional sense). Thus it is *contradictory* for the same participant to make both Intrusion and Fatalism errors (as it amounts to accepting both that “the agent would have done X no matter what” and “there was at least a slight chance that the agent might have done X”). But, 90% of participants perceived Fatalism and 57% perceived Intrusion. This means that *at least* 47% of participants perceived both Fatalism and Intrusion—which suggest that at least 47% of participants either did not interpret these measures the intended way or rejected the principle of noncontradiction. Being charitable (and thus assuming that participants do *not* reject the principle of noncontradiction), this suggests that (at least) one of these two measures (Intrusion or Fatalism) is not interpreted by participants in the intended way. A similar problematic pattern of answers can be found in the results of Murray and colleagues (forthcoming): they found that, on average, participants’ answers were above the mean for both Intrusion and Fatalism measures, which (again) is contradictory.

A second reason to doubt the validity of their measures come from recent studies examining these measures. Giraud and colleagues (2023) presented participants with scenarios featuring deterministic universes in which an agent is sent back in time repeatedly (thus experiencing the same day again and again). Participants were asked whether the same events (including human actions) would replay again and again and to fill an adapted version of Nadelhoffer and colleagues (2014)’ Determinism scale. Participants’ answers to both questions suggested that participants understood that the universe was deterministic. Still, when presented with Intrusion measures, they still obtained high Intrusion scores, and participants’ answers to Intrusion measures only correlated weakly with their scores to the Determinism scale (while both measures should be strongly negatively correlated if participants understood them as intended). Giraud and colleagues suggest that this might be due to participants’ interpreting Intrusion measures as being about the conditional rather than the unconditional ability to do otherwise. Similarly, Lim and colleagues (forthcoming) take issues with Murray and colleagues’ Intrusion measures: according to them, since these measures do not specify that the past should be held constant, it is not necessarily an error for participants to answer that agents in a deterministic universe have the possibility to act otherwise. Comparing Murray and colleagues’ Intrusion items to new items specifying that we should assume that the past remains the same, they found that their new items yielded significantly lower Intrusion error rates than Murray and colleagues’ original items.

Thus, it might be that participants understand the vignette but fail to interpret the Intrusion measures (and possibly other measures) in the intended way. We investigated this possibility in Studies 1–3.

(ii) Another reason to doubt the results of these research might come from the *samples* they have been collecting their data from. Indeed, to conclude that vignettes are too complex for participants to understand, one must first ensure that participants are doing their best. However, Nadelhoffer and colleagues (2020, 2023) and Murray and colleagues (forthcoming) recruited their participants on Amazon Mechanical Turk, without using any further recruitment tool to filter participants. This is worrisome because all these studies are fairly recent and several researchers have been warning about a recent drop in data quality on Amazon Mechanical Turk (Peer et al., 2022; Webb & Tangney, in press). Thus, the high rate of comprehension errors could be due to low quality data and inattentive participants.^3^ We investigated this possibility in Study 4.

The results of Studies 1–4 suggest that participants make much less intrusion errors than what has been claimed in the literature. However, this leaves us with Bypassing and Fatalism errors. This is why, in Study 5, we take the description of determinism used in Studies 1–4 and improve on it in order to decrease these other comprehension errors. We argue that, against (3) the results of this study provides additional reason for optimism, both because rates of comprehension errors can be reduced and because remaining errors are largely inconsequential.

## Open Science Statement

Materials, data, and analysis scripts for all studies are available at osf.io/vr9my/

## Study 1

In Study 1, our aim was twofold. First and foremost, we wanted to assess the criterion validity of Intrusion measures: do Intrusion measures really measure what they are supposed to measure (i.e. the *unconditional* ability to do otherwise)? To find out, we compared participants’ Intrusion scores with two other measures supposed to capture the same idea: Nadelhoffer and colleagues (2014)’s Determinism scale, and a question about what would happen if a certain event was replayed several times.

Secondly, we wanted to test the theory behind Intrusion scores by investigating whether participants’ Intrusion scores were actually driven by an indeterministic conception of human agency. Indeed, as we mentioned, the theory behind the hypothesis that intrusion errors might be systematic (and thus unavoidable) is that participants have an indeterminist view of human agency that prevents them to truly accept the idea that agents are determined in the vignettes used by experimental philosophers. One prediction that can be drawn from this theory is that intrusion errors should be specific to human agency and should not extend to other phenomena. Nadelhoffer and colleagues (2020) put this hypothesis to test by having participants compare a human agent and robot and found that participants indeed made much less intrusion errors in the case of the robot. However, this difference might be due not to participants having a special conception of human agency, but rather to their having a special conception of robotic agency. After all, *robots* are generally considered as a paradigmatic case of an entity deprived of any ability to do otherwise. Thus, in this study, we compared human agency to animal agency and to natural phenomena.

### Methods

219 United States residents recruited through Prolific Academic completed our survey. After exclusion based on two attention checks, we were left with 180 participants (90 men, 88 women, 2 others; *M*_age_ = 37.69, *SD*_age_ = 13.70). Participants were presented with the description of Universe A (deterministic) and Universe B (indeterministic) by Nichols and Knobe (2007), and asked which of these universes was most like ours.

They were then presented with three vignettes (in random order). Each vignette presented an event that took place in Universe A and described either a natural event (a *lightning* hitting a given tree out of three), an animal decision (a *cat* choosing in which basket to sleep out of three), or a human decision (a *woman* choosing a dessert out of three). Each vignette existed in two versions (one in which the most likely event happened: the lightning hitting the tallest tree or the woman choosing her favorite dessert, one in which the least likely event happened: the lightning hitting the smallest tree or the woman choosing her least favorite dessert). Participants were randomly assigned to one of the two versions. As there were no significant differences between these versions, we collapsed them for analysis in the results.

After each vignette, participants were asked to rate their agreement (on a scale from -3 = “Strongly disagree” with 3 = “Strongly agree”) with four Intrusion statements. For example, for the *Lightning* case, the statements were:

- The lightning could have hit Tree B or C even if everything (including the laws of nature) had been exactly the same prior to the storm.
- There was at least a slight chance that the lightning could have struck Tree B or C even if everything (including the laws of nature) had been exactly the same prior to the storm.
- It was open for the lightning not to strike Tree A at the exact moment the storm broke.
- The lightning could have struck another tree even though the fact that there was a storm was completely caused.

We counted as successful participants who obtained an average score below the midpoint (0).

For each vignette, participants were then asked to imagine that a time-traveler hopped back in time several times to watch the event replays, and were asked to indicate whether they believed that the same event would happen in *all* replays, or if something different would happen in *some* replays. Here is the question for the *Lightning* case:

*Imagine that, in Universe A, a time-traveler observed the lightning strike Tree A then decided to go back in time 10 min before the storm broke to see whether the lightning would always strike Tree A. Imagine that he did that hundreds and hundreds of times and never interfered with the storm and the events that led to its appearance. According to you, which of the following claims is more plausible:*

- No matter how many times the time-traveler goes back in time, the lightning will always strike Tree A (as long as the time-traveler does not interfere).
- If the time-traveler tries long enough, there will be times when the lightning does not strike Tree A (even if the time-traveler does not interfere).
- I don't know.

We counted as successful participants who chose the first option (“the lightning will always strike Tree A”).

At the end of the study, participants were presented with Nadelhoffer and colleagues (2014)’s Free Will and Determinism subscales. However, those were modified to probe participants’ beliefs about Universe A rather than about their own world, e.g. “In Universe A, everything that has ever happened had to happen precisely as it did, given what happened before”). We counted as successful participants who obtained an average score above the midpoint (0).

### Results

*Universe.* 87.8% of participants answered that Universe B was “most like ours”.

*Comparisons between the three cases*. Participants’ intrusion scores, determinism scores and answers to the time-travel question for each case (Lightning, Cat, Human) are presented in Table 1.

**Table 1:** Participants’ Intrusion scores and answers to the Time-travel question for each case, and to the Determinism subscale in general (Study 1). For continuous scores, we indicate the mean and standard deviation. Success and Failure rates for the Intrusion, Time-travel and determinism questions are presented

	Lightning	Cat	Human
Intrusion	− 1.39 (1.78) Success: 71.1% Failure: 28.9%	− 1.30 (1.91) Success: 70% Failure: 30.0%	− 1.42 (1.88) Success: 72.2% Failure: 27.8%
Time-travel	Success: 81.7% Failure: 18.3%	Success: 82.2% Failure: 17.8%	Success: 82.2% Failure: 17.8%
Free will subscale	− 1.83 (1.37) score > midpoint (0): 11.1%		
Determinism	1.83 (1.08) Success (score > midpoint): 93.3% Failure (score < or = midpoint): 6.7%		

*Assessing the criterion validity of Intrusion measures*. We had two benchmarks to assess the criterion validity of Intrusion measures: participants’ answers to the Time-travel question and participants’ answers to the Determinism scale (α = 0.80). The first question was whether these different measures would yield similar estimates of the percentage of participants’ adequately understanding determinism. For the Human case, two chi-square tests suggested that Intrusion measures yielded higher Failure estimates than the Time-travel question: Χ^2^(1) = 4.56, *p* = 0.033, and than the Determinism scale: Χ^2^(1) = 26.68, *p* < 0.001.

**Fig. 1:**
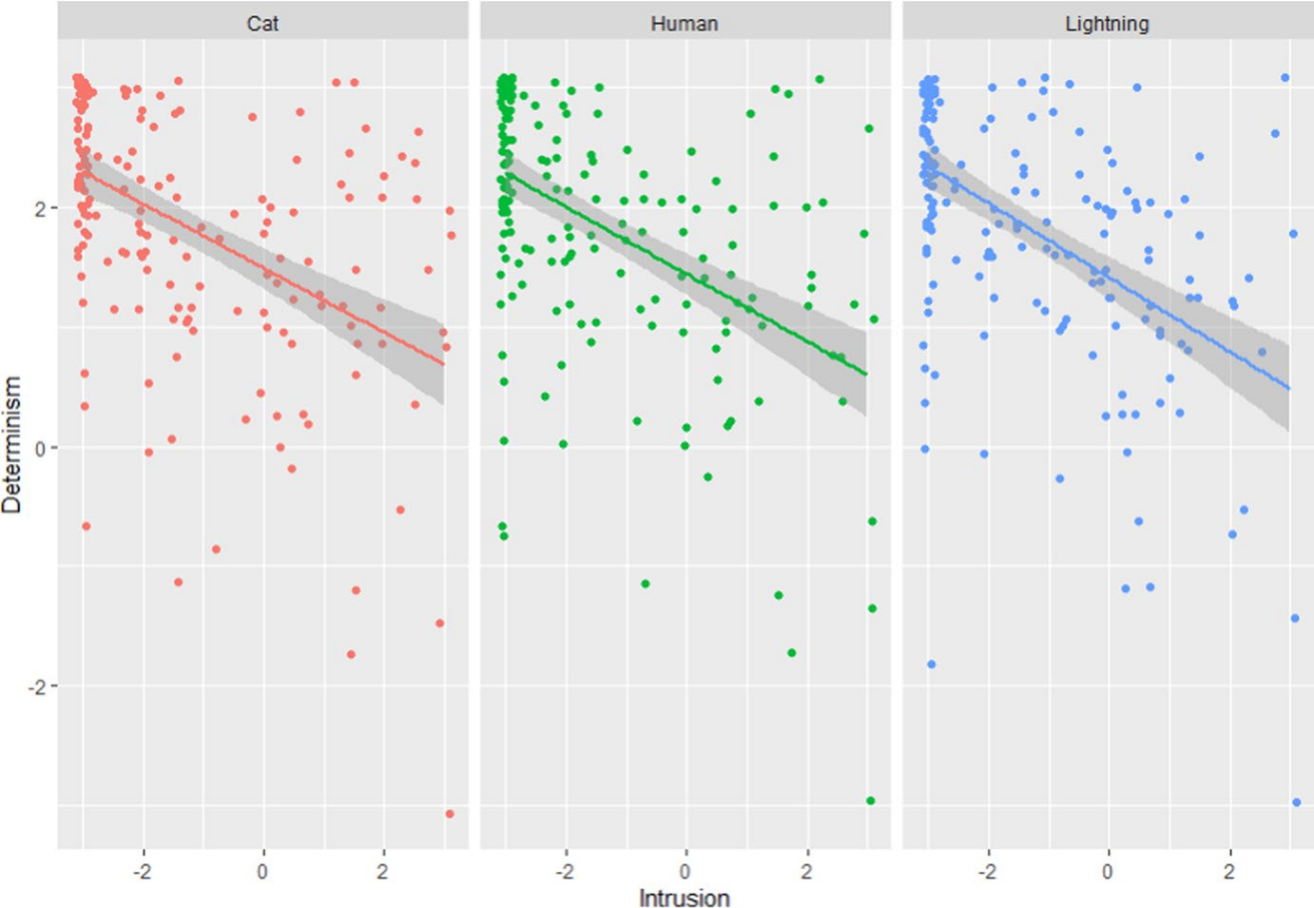
Determinism scores in function of Intrusion scores for each condition (Study 1)

The second question was whether participants’ answers to these different measures would be coherent, as they are supposed to measure similar or related constructs (indeed, belief in the type of ability to do otherwise that the Intrusion measures are supposed to measure are incompatible with the belief in determinism that the Determinism scale is supposed to measure). Since Intrusion measures and the Determinism scale were both continuous variables, we computed the extent to which they correlated with each other. We found significant correlations between participants’ answers to the Determinism scale and Intrusion scores for the Lightning (*r* = −0.51 [−0.61, −0.40], *p* < 0.001), Cat (*r* = -.−0.48 [−0.58, −0.36], *p* < 0.001), and Human cases (*r* = −0.49 [−0.59, −0.37], *p* < 0.001) (see Fig. 1). However, these correlations were only medium-sized. To compare participants’ answers to the Time-travel question with their answers to the Intrusion measures, we looked at the frequency with which both measures agreed on the fact that a participant succeeded or not. The two measures agreed for 144 participants out 180 (80%), which was significantly higher than what we could expect from chance: Χ^2^(1) = 35.10, *p* < 0.001.

**Fig. 2:**
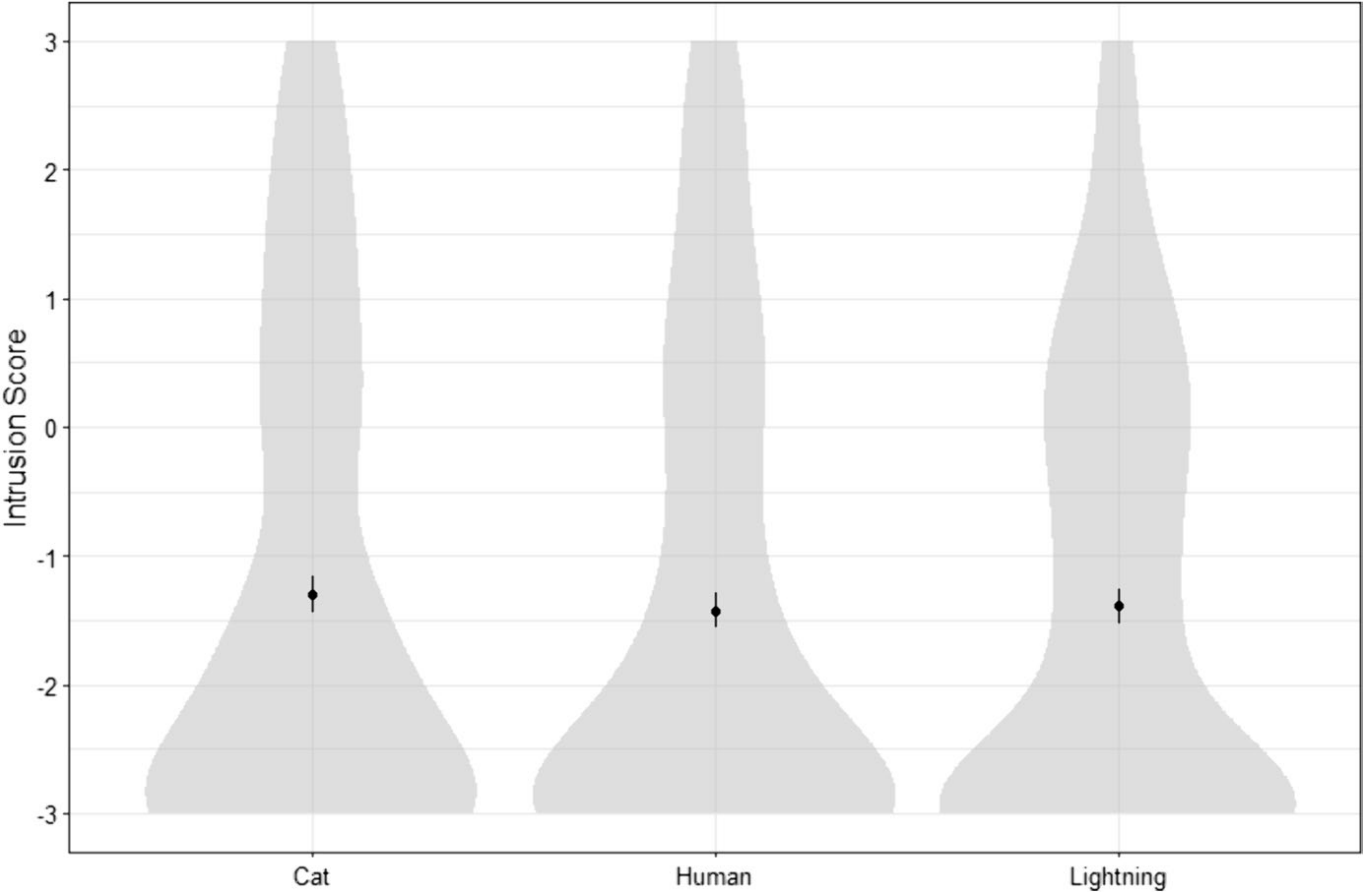
Distribution of participants’ Intrusion scores for all three cases in Study 1

*Comparison between three cases.* A within-subject ANOVA found no significant difference in Intrusion scores between conditions: *F*(2,358) = 0.93, *p* = 0.396. Participants were not more likely to fail to understand determinism in the case of humans than in the case of lightning or cats (see Fig. 2).

*Discussion.* Our results suggest that all three measures (Intrusion, Time-travel, Determinism) yielded roughly similar conclusions concerning participants’ understanding of determinism. However, of the three measures, Intrusion measures are the one that gave the highest estimate of failure to understand determinism. Still, our results provide no reason to think that Intrusion measures *grossly* overestimate participants’ failure to comprehend determinism compared to other measures.

Interestingly, we did not find that participants made more Intrusion errors in the Human case compared to the Lightning and Cat cases. These results suggest either that, *if* participants indeed have an indeterministic conception of human agency, this conception is not specific to human agency (and extends to natural events and animals) *or* that it is not what is driving participants’ answers to the Intrusion measures.

What is intriguing, though, is that most participants actually *succeeded* in understanding determinism (on all three measures). This seems to go against previous results suggesting that participants are unable to integrate the idea that human actions can be determined. What might explain this difference?

## Study 2

One possible difference between our first study and previous studies is that previous studies generally used morally-loaded actions (such as robbing a bank) while we used only a morally neutral choice (choosing between three desserts). In Study 2, we put this hypothesis to the test by comparing participants’ success to the Intrusion test through three contexts in which we asked them to think about the agent’s moral responsibility: a morally neutral action (baking a cake), a morally bad action (robbing a bank), and a morally good action (saving a child from drowning).

### Methods

320 United States residents recruited through Prolific Academic completed our survey. After exclusion based on two attention checks, we were left with 296 participants (145 men, 145 women, 6 others; *M*_age_ = 38.39, *SD*_age_ = 13.72).

The design was the same as in Study 1, except for the content of the vignettes we used. We designed three different cases focusing on an agent named Jeremy Hall: a neutral one, a morally bad one, and a morally good one (see Table 2). Participants were randomly presented with one of the three cases. Just after reading the case, they were asked to rate their agreement with the two following statements: “Jeremy deserves praise/credit for [performing the corresponding action]” and “Jeremy deserves blame for [performing the corresponding action].” After that, they were asked to answer the same Intrusion, Time-travel and Determinism questions as in Study 1.

**Table 2:** The three vignettes (Neutral, Bad, Good) used in Study 2

Neutral	Bad	Good
**In Universe A**, a man named Jeremy Hall is planning to bake a cake for himself. After checking the ingredients he has, he hesitates between baking a chocolate cake or a strawberry cheesecake. Because he loves chocolate, he decides to bake a chocolate cake. Acting on his decision, Jeremy bakes a chocolate cake then eats it	**In Universe A**, a man named Jeremy Hall dreams about buying an expensive car. Because he does not have enough money, Jeremy decides that the most efficient way to achieve his dream is to rob the nearest bank. Acting on his decision, Jeremy robs the bank and escapes with more than enough money to buy the car of his dreams	**In Universe A**, a man named Jeremy Hall is taking a walk along the countryside when he sees a child drowning into a shallow pond. Because there is no else besides him and the child, Jeremy decides that the morally right thing to do is to save the child. Acting on his decision, Jeremy walks into the pond and saves the child from drowning

**Fig. 3:**
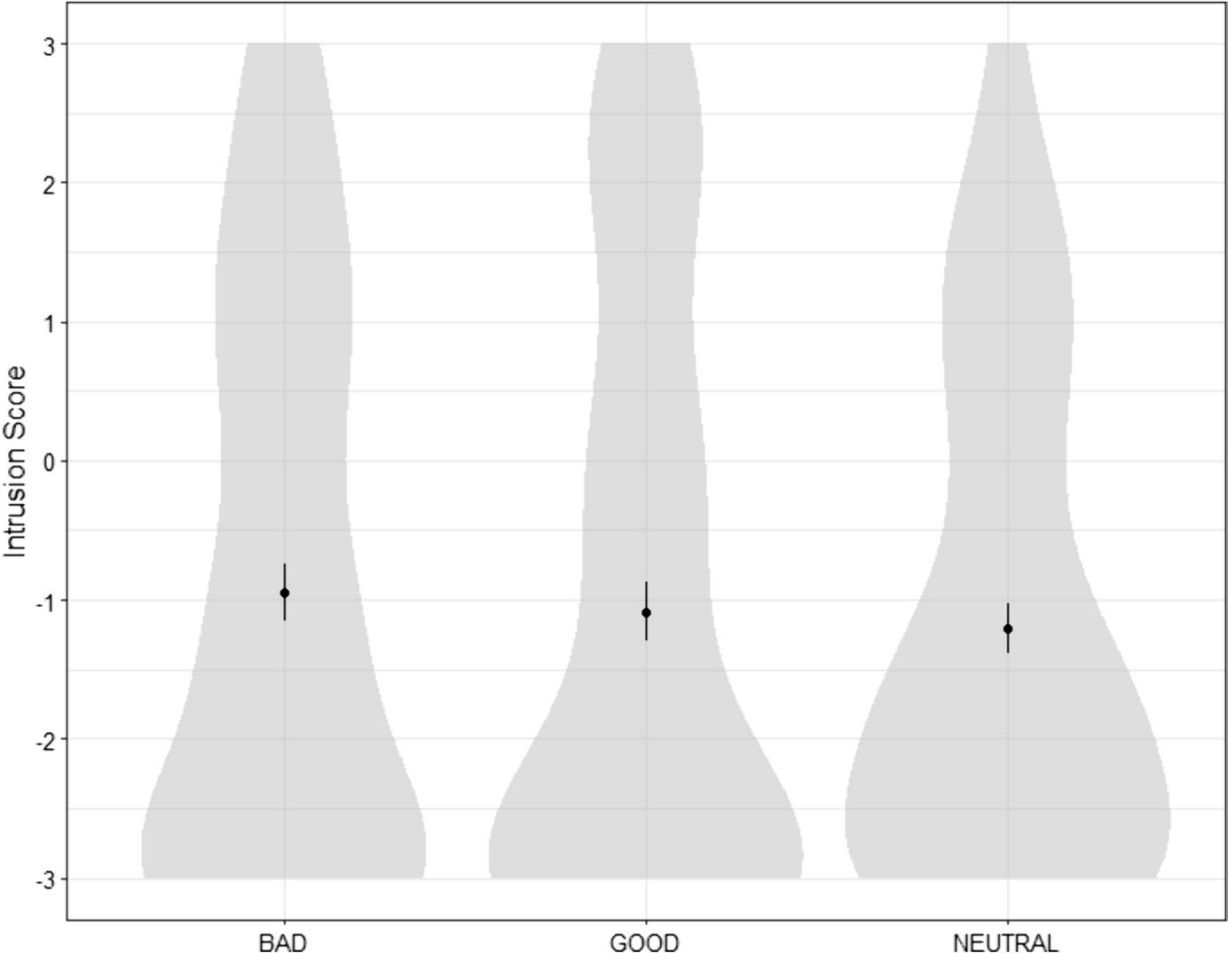
Distribution of participants’ Intrusion scores for all three cases in Study 2

### Results

*Universe.* 81.8% of participants answered that Universe B was “most like ours”.

*Comparisons between the three cases*. Participants’ intrusion scores, determinism scores and answers to the blame, praise, and time-travel question for each case (Neutral, Bad, Good) are presented in Table 3 and Fig. 3 Contrary to our initial hypothesis, a between-subject ANOVA found no significant difference in Intrusion between the three cases: *F*(2, 293) = 0.40, *p* = 0.674.

**Table 3:** Participants’ Intrusion and Determinism scores and answers to the Blame, Praise and Time-travel questions for each case (Study 2). For each continuous variable, we indicate the mean, the standard deviation and the % of participants who obtained a score superior to the midpoint (= 0). Superscript letters indicate the results of a comparison of all three cases using a post-hoc Tukey test: conditions that share a common letter do not differ significantly from each other (*p* < .05)

	Neutral	Bad	Good
Blame	− 1.20^b^ (1.71) 15.0%	0.62^c^ (2.15) 50.5%	− 2.46^a^ (1.33) 5.1%
Praise/Credit	0.05^b^ (1.82) 36.0%	− 1.86^a^ (1.53) 7.2%	1.85^c^ (1.84) 80.8%
Intrusion	− 1.21^a^ (1.79) Success: 71.0% Failure: 29.0%	− 0.95^a^ (2.03) Success: 64.7% Failure: 35.3%	− 1.09^a^ (2.12) Success: 66.6% Failure: 33.3%
Determinism	1.69^a^ (1.03) Success: 93.0% Failure: 7.0%	1.75^a^ (1.00) Success: 93.8% Failure: 6.2%	1.76^a^ (1.04) Success: 91.9% Failure: 8.1%
Time-travel	Success: 88.0%^a^ Failure: 12.0%	Success: 82.5%^a^ Failure: 17.5%	Success: 92.9%^a^ Failure: 7.1%
Free Will subscale	− 1.38^a^ (1.45) 20.0%	− 1.43^a^ (1.47) 17.5%	− 1.54^a^ (1.45) 18.1%

*Criterion validity of Intrusion measures.* As for Study 1, two Chi-square tests showed that Intrusion measures yielded significantly higher estimates of failure to understand determinism compared to the Time-travel question: Χ^2^(1) = 34.91, *p* < 0.001, and to the Determinism scale: Χ^2^(1) = 59.54, *p* < 0.001.

**Fig. 4:**
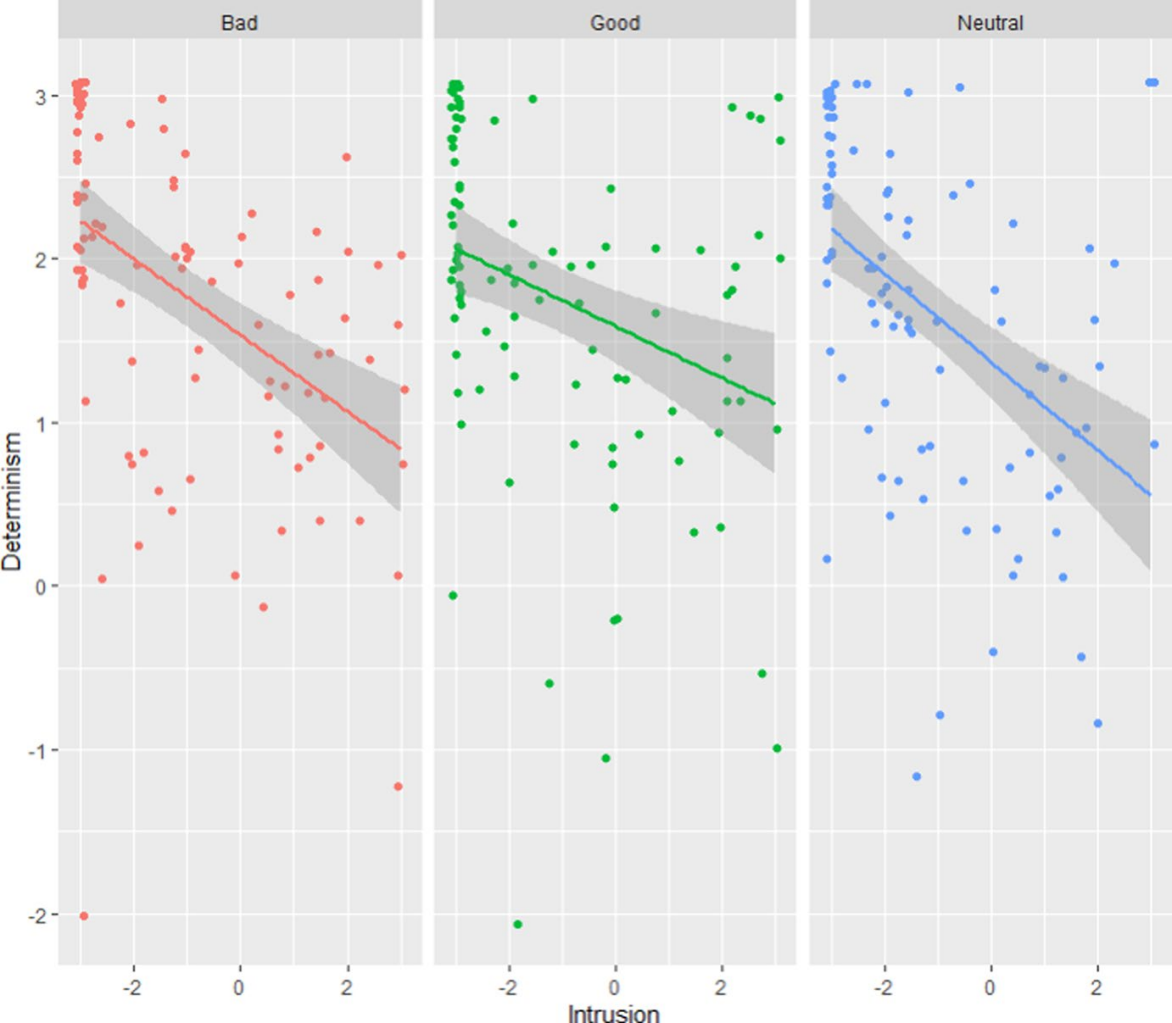
Determinism scores in function of Intrusion scores for each condition (Study 2)

Correlations between Intrusion and Determinism scores were significant, but below the standard threshold for medium-sized correlations: *r* = −0.41 [−0.50, -0.32], *p* < 0.001. Intrusion measures (see Fig. 4). When it came to estimating success or failure to understand determinism, Intrusion measures and the Time-travel questions agreed for 213 participants out of 296 (72%), which was significantly different from chance: Χ^2^(1) = 23.16, *p* < 0.001.

*Additional analyses*. Despite the fact that most participants seemed to understand that agents did not have the unconditional ability to do otherwise in Universe A, most attributed praise to the agent in the Good case, and half of them attributed blame to the agent in the Bad case. In the Bad case, Intrusion scores correlated significantly with Free Will scores (*r* = 0.65, *p* < 0.001) and Blame attributions (*r* = −0.21, *p* = 0.041). In the Good case, Intrusion scores correlated significantly with Free Will scores (*r* = 0.54, *p* < 0.001), but not with Praise attributions (*r* = −0.05, *p* = 0.647). The difference between the Bad and the Good case could be explained by the fact that participants might be less likely to confuse determinism with bypassing in the Good case, since they are generally less reluctant to attribute morally good desires to agents (Cova, 2022). Moreover, the fact that participants still attribute blame and praise while obtaining low scores on the Free Will scale might suggest that the Free Will scale (and, more generally, all measures that directly ask participants about “free will”) does not capture the kind of free will relevant to moral responsibility and that is of interest to most philosophers in the contemporary debate (Cova, 2023). Indeed, Free Will scores did not significantly correlate with Blame attributions for the Bad case (*r* = −0.09, *p* = 0.403) or Praise attributions for the Good case (*r* = 0.06, *p* = 0.542). For these reasons, we will focus on measures of moral responsibility (blame and praise) rather than on measures on free will in the following sections.

*Discussion.* As in Study 1, we found that Intrusions measures reliably tracked our two other measures of comprehension of determinism, while yielding significantly higher estimates of failure to comprehend determinism.

However, we once again found that, on all three measures, most participants seemed to understand determinism. Using (negatively or positively) morally loaded scenarios did not seem to have any impact.

## Study 3

One possibility is that our participants only *seem* to understand determinism. To investigate to what extent their answers to our measures of comprehension of determinism actually reflect a genuine understanding of determinism, we decided to manipulate the description of the world (either a deterministic or indeterministic world) and see whether participants’ answers to these measures would vary accordingly. As such, in Study 3, we ran a study similar to the one presented by Nichols and Knobe (2007), in which we asked participants either about a deterministic or an indeterministic universe. Additionally, we asked participants to justify their answers and analyzed their justifications to detect possible misunderstandings.

### Methods

797 United States residents recruited through Prolific Academic completed our survey. After excluding participants who failed at least one of the two attention checks, we were left with 764 participants (385 men, 366 women, 13 others; *M*_age_ = 38.52, *SD*_age_ = 15.24).

Participants were first presented with Nichols and Knobe’s descriptions of Universe A and B and asked which of these two universes were more like ours. They were then randomly assigned to one of four conditions: *abstract case in Universe A*, *abstract case in Universe B, concrete case in Universe A,* and *concrete case in Universe B*. Participants in the *concrete* conditions were presented with a small vignette telling them the story of Bill, an agent living in the corresponding universe, who kills his wife and three children to be with his secretary (see Nichols & Knobe, 2007).

Participants in the *abstract* conditions were asked to which extent they agreed with the following statements (on a scale from −3 to 3): “In Universe A, it is possible for people to be morally responsible for their action”, “In Universe A, people deserve to be blamed for the bad things they do”, “In Universe A, it is possible for people to have free will”, and “In Universe A, people's decisions are *up to them*”. Participants in the *concrete* conditions were asked to which extent they agreed with the following statements: “Bill is morally responsible for killing his family”, “Bill deserves to be blamed for killing his family”, “Bill has free will”, and “Bill's decision to kill his family was *up to him*”. Participants in both conditions were asked to justify their answer to the moral responsibility question.

After that, participants were asked to rate their agreement with 10 statements. 4 statements were inspired from Murray and Nahmias (2014) and measured participants’ perception of *bypassing*. Their formulation varied between the abstract and concrete conditions (e.g. “In Universe A, people's decisions have no effect on what they end up doing” vs. “Bill's decision to kill his family had no effect on what he ended up doing”). 2 statements measured participants’ perception of whether agents’ action can stem from their *deep self* and their formulation varied across conditions. 4 statements were inspired from the *intrusion* statements used by Nadelhoffer and colleagues. Their formulation was the same in both abstract and concrete conditions (e.g. “In Universe A, what people decide to do could have been different even if everything leading up to the decision had been exactly the same”).

Participants were then asked a Time-travel question about the Universe they were asked about (A or B). They were told that a time-traveler observed John’s decision to have French fries and then went back in time a hundred times to see whether John would always choose French fries. Participants were asked the number of observations (out of 100) on which John would choose to have French fries. They had to choose a number between 0 and 100.

Finally, participants had to answer two attention checks, fill a small personality questionnaire (Gosling et al., 2003), and provide information about them (age and gender).

### Results

Main results are presented in Table 4.

**Table 4:** Mean and standard deviations for participants’ answers to the various questions asked in Study 3, for each case (abstract vs. concrete) and each sample (A vs. B). % indicate the percentage of answers superior to the midpoint, except when some other cutoff is specified. Superscript letters indicate the results of a comparison of all three cases using a post-hoc Tukey test: conditions that share a common letter do not differ significantly from each other (*p* < .05)

	Universe A		Universe B	
	Abstract	Concrete	Abstract	Concrete
Responsibility	− 0.90^a^ (2.09) 27.9%	1.21^b^ (2.30) 67.2%	2.38^c^ (0.94) 94.8%	2.74^c^ (1.06) 95.4%
Blame	− 0.99^a^ (2.00) 10.5%	1.35^b^ (2.19) 70.4%	1.83^c^ (1.29) 86.9%	2.95^d^ (0.21) 100%
Free will	− 1.56^a^ (1.86) 18.4%	− 0.24^b^ (2.51) 44.1%	2.38^c^ (0.82) 96.3%	2.74^c^ (0.71) 97.5%
Bypassing	0.83^d^ (1.80) < 0: 28.9%	− 0.79^c^ (1.85) < 0: 65.0%	− 1.75^b^ (1.22) < 0: 88.5%	− 2.40^a^ (0.91) < 0: 97.5%
Deep self	− 1.10^a^ (1.67) < 0: 15.3%	0.74^b^ (1.74) < 0: 56.5%	1.63^c^ (1.10) < 0: 84.3%	2.32^d^ (1.05) < 0: 91.9%
Intrusion	− 2.05^a^ (1.35) < 0: 89.5%	− 1.26^b^ (1.89) < 0: 69.4%	2.29^c^ (0.76) < 0: 0.5%	2.48^c^ (0.86) < 0: 3.1%
Time-travel	92.58^a^ (20.39) = 100: 78.9%	91.99^a^ (19.82) = 100: 74.7%	70.12^b^ (23.99) = 100: 20.4%	70.73^b^ (23.53) = 100: 21.8%

**Fig. 5:**
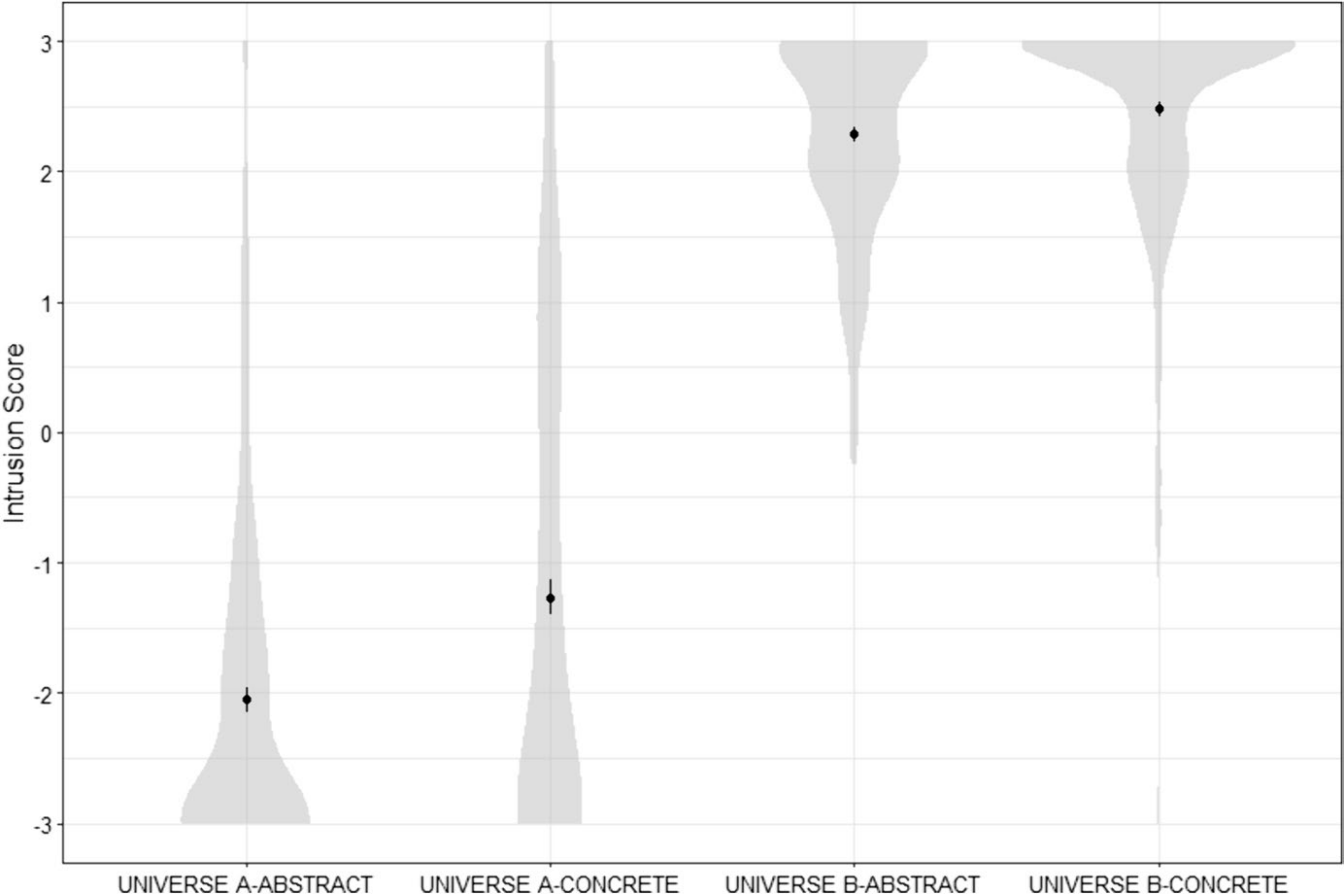
Distribution of participants’ Intrusion scores for all four conditions in Study 3

*Effect of manipulation on measures of comprehension of determinism.* An ANOVA with Intrusion scores as dependent variable and Universe (A or B) and Condition (Abstract or Concrete) as factors found a significant effect of Universe: *F*(1,760) = 1885.94, *p* < 0.001, a significant effect of Condition: *F*(1,760) = 27.11, *p* < 0.001, and a significant interaction effect: *F*(1,760) = 10.14, *p* = 0.002. The interaction effect is due to the fact that there was a significant difference between the Abstract and Concrete conditions in Universe A: *t*(334.58) = 4.64, *p* < 0.001, and but no significant difference between the Abstract and Concrete conditions in Universe B: *t*(382.97) = 2.34, *p* = 0.020 (see Fig. 5).

An ANOVA with answers to the Time-travel question as dependent variable and Universe (A or B) and Condition (Abstract or Concrete) as factors found a significant effect of Universe: *F*(1,760) = 185.42, *p* < 0.001, but no significant effect of Condition: *F*(1,760) = 0.01, *p* = 0.916, and no significant interaction effect: *F*(1,760) = 0.08, *p* = 0.778.

Overall both measures were sensitive to the type of Universe described in the vignette: participants gave answers consistent with the type of Universe they were presented with. However, one difference between the two measures of comprehension of determinism was that Intrusion scores were sensitive to Condition (Abstract vs. Concrete) while answers to the Time-travel question were not. This suggests that Intrusion measures do not *only* measure participants’ perception of the unconditional ability to do otherwise but also something else. In line with previous suggestions by Giraud and colleagues (2023), our interpretation is that some participants interpret Intrusions items as being about the unconditional ability to do otherwise, while others interpret them as being about the *conditional* ability to do otherwise. As evidenced by Bypassing scores (and in line with the previous literature), participants are more likely to attribute the conditional ability to do otherwise (that is: the ability to act according to one’s desires and beliefs) in the Concrete version of Universe A than in the Abstract version of Universe A. Given that Intrusion scores increase in the Concrete version compared to the Abstract one, this suggests that some participants might interpret Intrusion items as measuring the very type of control that is prevented by Bypassing. This interpretation of the data is supported by the fact that Intrusion measures showed significant correlations with answers to the Bypassing (*r* = −0.66, *p* < 0.001) and Deep Self scores (*r* = 0.67, *p* < 0.001).

*Criterion validity of Intrusion measures.* There was a significant, though moderate-sized, correlation between Intrusion scores and participants’ answers to the Time-travel question: *r* = −0.45, *p* < 0.001. For Universe A (the deterministic universe), both measures agreed on the fact that participants successfully understood determinism in 298 cases out of 376 (79.3%). This was significantly different from chance: Χ^2^(1) = 55.95, *p* < 0.001.

*Justifications*. We performed a qualitative analysis of participants’ justifications for their answers to the Responsibility question. The whole procedure is detailed in Supplementary Materials. Overall, we did not find convincing evidence that participants imported indeterministic assumptions in their understanding of the vignettes: among participants who attributed moral responsibility to an agent in a deterministic universe (Universe A), very few explicitly rejected deterministic assumptions (0% in the Abstract case, 1.6% in the Concrete case). This provides additional reasons to think that most participants do not fail to abide by the deterministic assumptions of such vignettes.

*Additional analyses*. To investigate which type of factors best predicted participants’ attributions of moral responsibility, we conducted a multiple regression analysis with participants’ answers to the Moral Responsibility question, and Intrusion, Bypassing, Deep Self, and Time Travel answers as predictors. Results are presented in Table 5.

**Table 5:** Results of multiple regression analysis with Responsibility scores as dependent variable and Bypassing, Deep Self, Intrusion, and Time-travel (binary) scores as predictors (Study 3). The analysis is performed twice: a first time with answers to both Universes A and B pooled together, and a second time with only answers to Universe A. The *r* column indicates zero-order correlations between each factor and Responsibility scores

	*r*	B	β	SE	t	*p*
(a) Universes A and B						
Intercept	–	0.70	–	0.09	7.710	< .001***
Bypassing	− 72***	− 0.45	− 0.39	0.05	− 9.557	< .001***
Deep Self	.68***	0.20	0.18	0.05	4.274	< .001***
Intrusion	.67***	0.25	0.27	0.03	7.212	< .001***
Time travel	− 43***	− 0.17	− 0.04	0.13	− 1.298	0.195
*R*^*2*^ = .596 (*R*^*2*^ = .585 with only Bypassing and Intrusion as predictors)						
(b) Universe A						
Intercept	–	0.95	–	0.19	4.968	< .001***
Bypassing	− 67***	− 0.52	− 0.42	0.07	− 7.125	< .001***
Deep Self	.62***	0.23	0.18	0.07	3.062	0.002**
Intrusion	.50***	0.278	0.19	0.07	4.238	< .001***
Time travel	− 30***	− 0.37	− 0.06	0.24	− 1.56	0.121
*R*^*2*^ = .512 (*R*^*2*^ = .497 with only Bypassing and Intrusion as predictors)						

*Discussion.* As in Studies 1 and 2, participants’ answers to the Intrusion measures and the Time-travel question suggest that most of them understood that Universe A was deterministic. Moreover, the fact that their answer varied depending on which Universe they were presented with (A or B) suggests that their answer reflected a genuine understanding of the vignettes. Qualitative analysis of their justification to the Responsibility questions suggest that very few of them actually appealed to indeterministic conceptions of agency in Universe A. Overall, the results of Study 3 suggests that most participants are successful in understanding when the universe described is deterministic or not (though other errors, such as the fact that Universe A involves bypassing in addition to determinism were pervasive, particularly in the Abstract case).

As in Studies 1 and 2, our two measures of comprehension of determinism (Intrusions scores and the Time-travel question) yielded roughly similar verdicts. However, there was a difference between the two measures: while participants’ answers to the Time-travel question were only sensitive to the Universe described (A or B), Intrusion measures were also sensitive to the Condition (Abstract or Concrete). This suggests that Intrusion measures might not *only* measure participants’ perception of determinism and the unconditional ability to do otherwise, but also different constructs.

## Study 4

Through Studies 1–3, we observed that most participants succeeded at the Intrusion test, as well at similar tests such as the Time-travel test and Determinism test. This is very different from recent results, such as the ones obtained by Nadelhoffer and colleagues (2023) and Murray and colleagues (forthcoming). However, it does not seem that such differences can be explained by differences in materials or methods. Thus, in Study 4, we turn to a different possibility: that the difference might be due to differences in population we draw our samples from. Indeed, while all our participants were recruited from Prolific Academic, Nadelhoffer and colleagues (2023) and Murray and colleagues (forthcoming) recruited theirs from Amazon Mechanical Turk.

This might seem a very *ad hoc* hypothesis, but there are reasons to think that there might be something special about the samples used in these studies. Indeed, when presented with Nichols and Knobe’s description of determinism and asked which Universe (A or B) was more like ours, Nadelhoffer and colleagues (2023) found that participants were very split on this question. Murray and colleagues (forthcoming) report similar results. This pattern of responses is at odds not only with the one we observed (where most participants answer “Universe B”), but with the whole literature using this paradigm (Nichols & Knobe, 2007; Sarkissian et al., 2010). This suggests that there might be something odd with the samples used by Nadelhoffer and colleagues (2023) and Murray and colleagues (forthcoming).

We thus decided to directly compare data collected from both populations.

### Methods

We contacted Nadelhoffer and colleagues to make sure that our design would mirror their design as closely as possible, especially when it came to participants’ selection.

Participants were recruited both from Amazon Mechanical Turk and Prolific Academic at the exact same time. For both sources, only United States residents were allowed to participate. For Mechanical Turk, only participants who had already completed at least 500 tasks in the past and had received an approval rate of 97% or more were allowed to participate. There was no such restriction for participants recruited on Prolific Academic.

As a first filter, participants were asked to prove they were not a bot by answering two questions: “Are you a robot” (YES/NO) and “Monday is the first day of the week. What is the third day of the week?” (participants answered by selecting a day of the week in a 7-option list).

Then participants were presented with the same description of Universes A and B as in previous studies. Just after, they were asked which of these universes is the most similar to ours (Universe A or Universe B), then were presented with what we will call the ‘obvious’ comprehension question:

The following question is designed to make sure you properly understood the scenario:

In Universe A, everything that happens is completely caused by what happened before it. True or false? (TRUE/FALSE).

In line with Nadelhoffer and colleagues’ original design, participants were also asked whether they thought Universe A was possible (Yes/No) and how vividly they could imagine the previously described scenario (on a 5-point scale from “Very slightly or not all” to “Extremely”).

After exclusion based on the two bot checks, 299 participants from Mechanical Turk (MTurkers) and 242 participants from Prolific Academic (Prolificers) completed our survey. Out of 299 MTurkers, 287 succeeded at the obvious comprehension check (174 men, 112 women, 1 other; *M*_age_ = 36.37, *SD*_age_ = 10.14). Out of 242 Prolificers, 238 succeeded at the obvious comprehension check (125 men, 112 women, 1 other; *M*_age_ = 36.74, *SD*_age_ = 14.14).

Participants were then randomly assigned to the *abstract* or *concrete* condition (for exploratory purposes, a third of participants recruited from Prolific were assigned to a *morally good concrete* condition (see Supplementary Materials). Participants in the abstract condition were asked three questions about the person living in Universe A: whether they can be morally *praiseworthy* when they do something right, whether they can be morally *blameworthy* when they do something wrong, and whether it is possible for them to have *free will*. Participants in the concrete condition were presented with the case of Jeremy robbing a bank (see Study 2) then asked three questions about Jeremy: whether he deserved *praise* for robbing the bank, whether he deserved *blame* for robbing the bank, and whether he robbed the bank of his *own free will*. Participants answered all questions on a 7-point scale (from -3: “Strongly disagree” to 3: “Strongly agree”).

Participants were then asked to rate their agreement with a list of 14 statements. 4 statements were inspired from the Nadelhoffer and colleagues’ Intrusion measure, 4 from their Epiphenomenalism measure, and 4 from their Fatalism measure. In the abstract condition, items were formulated to be about John (the man eating fries in the description of Universe A) while, in the concrete condition, they were formulated to be about Jeremy. The two additional items were ‘discreet’ attention checks (in the sense that they were not explicitly flagged as attention check):

- Check A: [John/Jeremy] lives in Universe A
- Check B: [John/Jeremy] lives in Universe B

After that participants were asked the same Time-travel question as in Study 3 (and to give an answer between 0 and 100). For the abstract condition, the question was about John deciding to have fries. For the concrete condition, the question was about Jeremy deciding to rob the bank.

Participants were also presented with the same modified Determinism scale as in Studies 1 and 2 and asked to indicate their age and gender.

### Results

Participants’ answers to all questions are presented in Table 6.

**Table 6:** Mean and standard deviations for participants’ answers to the various questions asked in Study 4, for each case (abstract vs. concrete) and each sample (MTurkers vs. Prolificers)

	Abstract		Concrete	
	MTurk	Prolific	MTurk	Prolific
Blame	1.06^b^ (1.63) 75.2%	− 0.19^a^ (1.95) 39.8%	1.62^c^ (1.27) 88.4%	1.08^bc^ (2.25) 64.2%
Praise	1.06^c^ (1.64) 73.0%	− 0.08^b^ (1.93) 38.9%	0.18^b^ (2.13) 55.5%	− 2.39^a^ (1.22) 2.5%
Free will	0.82^bc^ (1.90) 69.5%	− 1.31^a^ (1.69) 16.1%	1.23^c^ (1.63) 78.1%	0.24^b^ (2.40) 48.3%
Epiphenomenalism	1.43^ab^ (1.16) Success: 9.9% Failure: 90.1%	1.71^b^ (1.35) Success: 10.2% Failure: 89.8%	1.15^a^ (1.08) Success: 8.2% Failure: 91.8%	1.14^a^ (1.53) Success: 18.3% Failure: 81.7%
Fatalism	1.41^a^ (1.07) Success: 6.4% Failure: 93.6%	1.50^a^ (1.19) Success: 8.5% Failure: 91.5%	1.28^a^ (1.07) Success: 8.9% Failure: 91.1%	1.32^a^ (1.34) Success: 14.2% Failure: 85.8%
Intrusion	0.65^b^ (1.74) Success: 24.1% Failure: 75.9%	− 1.65^a^ (1.33) Success: 76.3% Failure: 23.7%	0.51^b^ (1.70) Success: 19.2% Failure: 80.8%	− 1.27^a^ (1.67) Success: 73.3% Failure: 26.7%
Time-travel	71.45^a^ (22.82) Success: 23.4% Failure: 76.6%	92.47^b^ (19.17) Success: 79.7% Failure: 20.3%	71.12^a^ (23.20) Success: 25.3% Failure: 74.7%	89.3^b^ (24.91) Success: 75.0% Failure: 25.0%
Determinism	1.52^ab^ (0.99) Success: 90.1% Failure: 9.9%	1.95^b^ (1.09) Success: 95.8% Failure: 4.2%	1.51^a^ (0.97) Success: 92.5% Failure: 7.5%	1.85^b^ (1.22) Success: 91.7% Failure: 8.3%
Check A	1.73^a^ (1.35) Success: 80.1% Failure: 19.9%	2.08^ab^ (1.52) Success: 81.4% Failure: 18.6%	1.66^a^ (1.52) Success: 82.2% Failure: 17.8%	2.50^b^ (1.33) Success: 91.7% Failure: 8.3%
Check B	0.10^b^ (2.18) Success: 37.6% Failure: 62.4%	− 1.83^a^ (1.89) Success: 72.0% Failure: 28.0%	− 0.18^b^ (2.20) Success: 41.8% Failure: 58.2%	− 2.43^a^ (1.43) Success: 90.8% Failure: 9.2%
*N*	141	118	146	120

% indicate the percentage of answers superior to the midpoint (when nothing else is indicated). Superscript letters indicate the results of a comparison of all three cases using a post-hoc Tukey test: conditions that share a common letter do not differ significantly from each other (*p* < .05)

*Universe*. 66.6% of MTurkers answered that Universe A was most like ours, while only 11.8% of Prolificers answered that Universe A was most like ours.

*Differences between samples in participants’ understanding of determinism.* An ANOVA with Intrusion scores as dependent variable and Sample (MTurk vs. Prolific) and Condition (Abstract vs. Concrete) as between-subject factors found a significant effect of sample: *F*(1,521) = 190.46, *p* < 0.001, no significant effect of condition:* F*(1,521) = 0.45, *p* = 0.501, and a marginally significant interaction effect: *F*(1,521) = 3.10, *p* = 0.079.

An ANOVA with participants’ answers to the Time-travel question as dependent variable and Sample (MTurk vs. Prolific) and Condition (Abstract vs. Concrete) as between-subject factors found a significant effect of sample: *F*(1,521) = 97.12, *p* < 0.001, no significant effect of condition:* F*(1,521) = 0.67, *p* = 0.413, and no significant interaction effect: *F*(1,521) = 0.51, *p* = 0.477.

An ANOVA with participants’ answers to the Determinism scale as dependent variable and Sample (MTurk vs. Prolific) and Condition (Abstract vs. Concrete) as between-subject factors found a significant effect of sample: *F*(1,521) = 17.13, *p* < 0.001, no significant effect of condition:* F*(1,521) = 0.29, *p* = 0.588, and no significant interaction effect: *F*(1,521) = 0.26, *p* = 0.607.

**Fig. 6:**
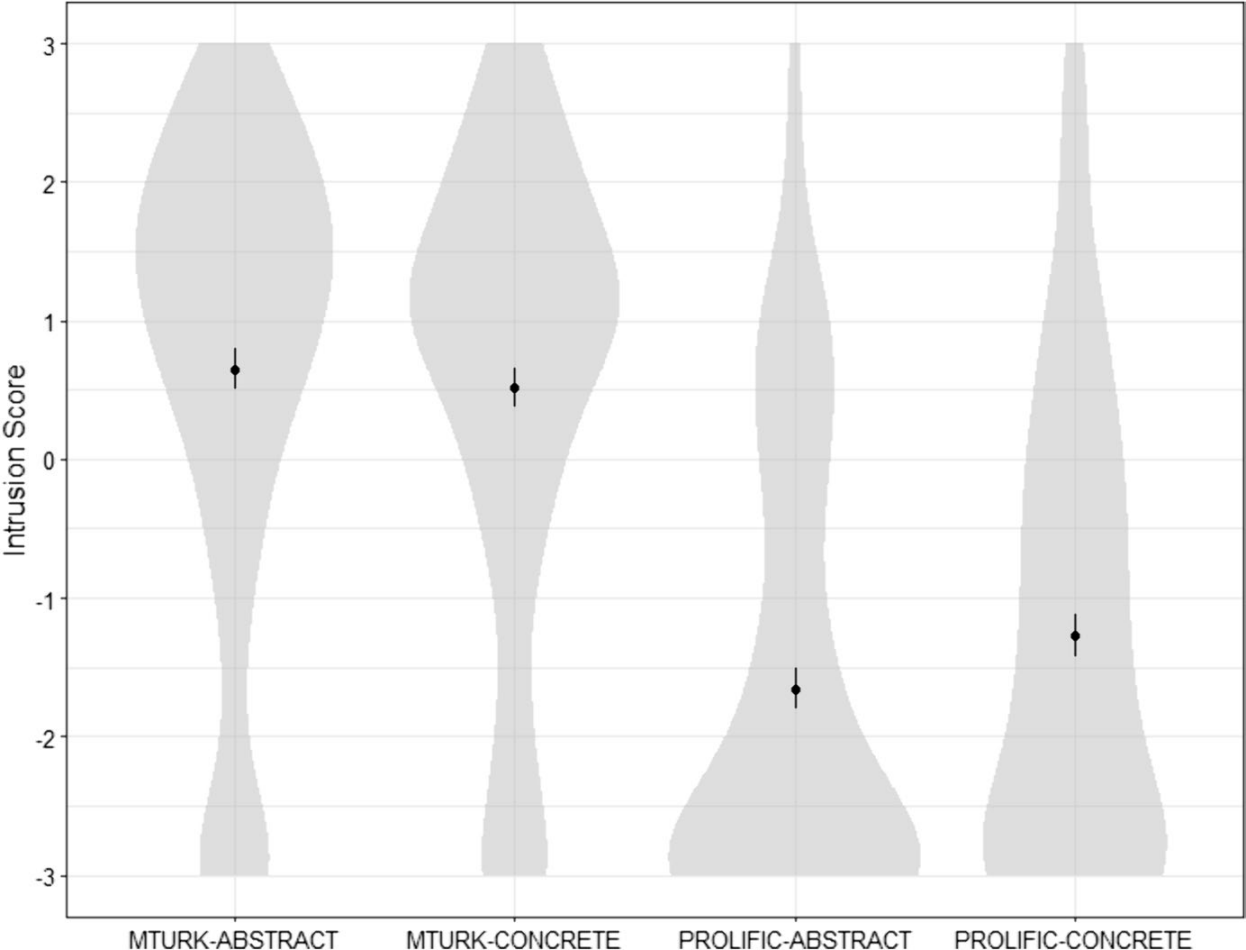
Distribution of participants’ Intrusion scores for all four conditions in Study 4

This means that, on all three measures, there were significant differences between MTurkers and Prolificers, with Prolificers tending to have better scores. As can be seen in Table 6 and Fig. 6, very few MTurkers (around 25%) succeeded at the Intrusion test, while most Prolificers (around 75%) succeeded. Similarly, MTurkers tended to fail the Time-travel test (around 25% of success), while most Prolificers succeeded (around 75%). However, both MTurkers and Prolificers succeeded at the Determinism test (> 90%). How can we explain this pattern of answer?

*Comparison of data quality.* A first cue can be found in the observation that MTurkers provided more nonsensical answers than Prolificers.

**Fig. 7:**
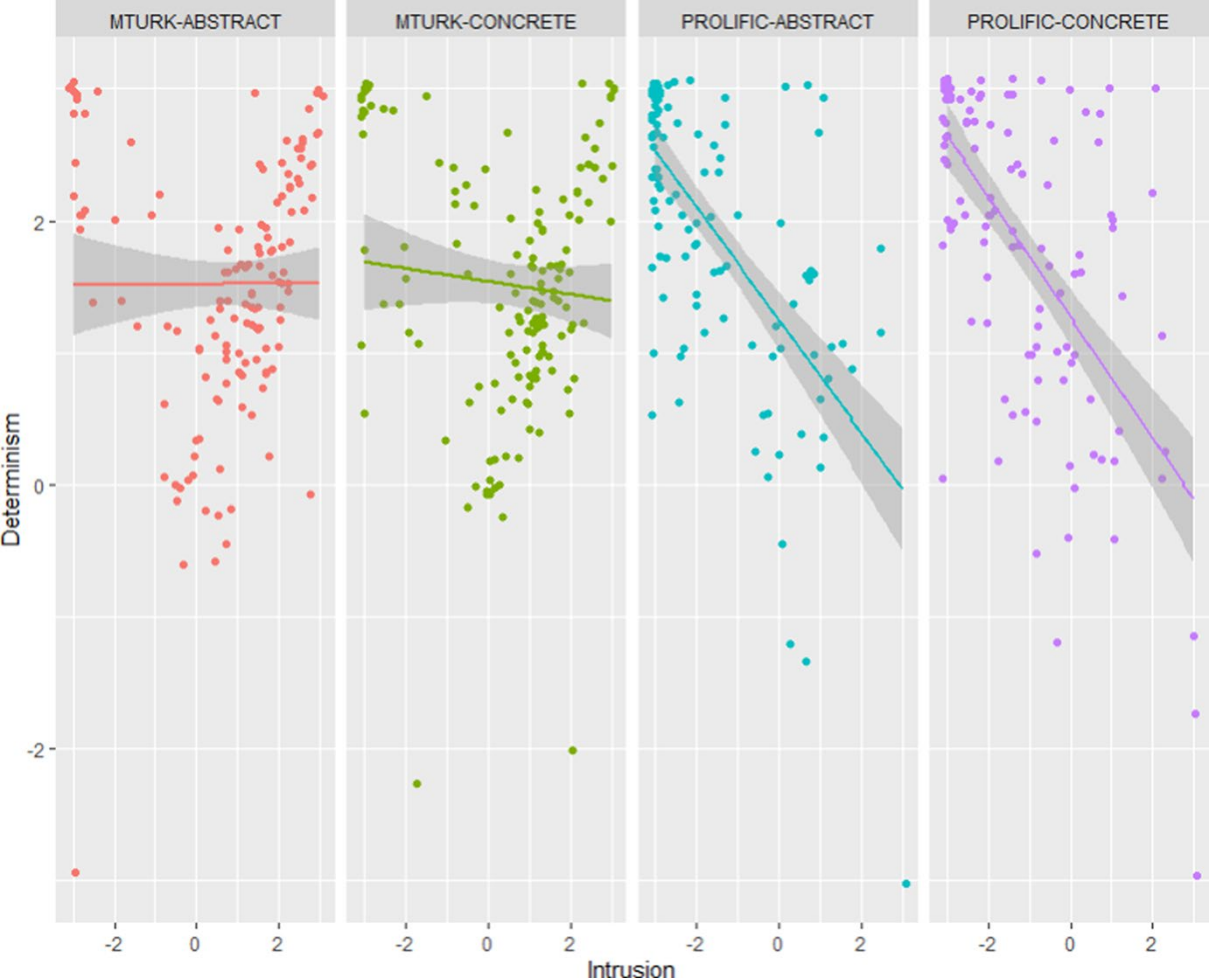
Determinism scores in function of Intrusion scores for each condition and sample (Study 4)

(i) We included in the study two “discreet” attention checks: Checks A and B. Two Welch t-tests show that Prolificers were significantly better than MTurkers on Check A: *t*(505.24) = 4.77, *p* < 0.001, and on Check B: *t*(520.6) = 12.33, *p* < 0.001.
(ii) The concrete case described someone robbing a bank. Still, 55% of MTurkers answered that Jeremy deserved *praise* for robbing the bank. One might object that MTurkers might have some very peculiar anti-capitalist morality. But 88.4% of MTurkers also answered that Jeremy deserved *blame* for robbing the bank. Thus means that at least 43% of them gave *inconsistent* answers.
(iii) One more reason to think that MTurkers’ pattern of answers make absolutely no sense is that they tend to agree with both the Fatalism and Intrusion statements, while these statements are incompatible and contradictory. For Prolificers, we find an inverse correlation between Fatalism and Intrusion scores: *r* = −0.56 [−0.65, −0.48], *p* < 0.001, while there is no such inverse correlation for MTurkers: *r* = 0.02 [−0.10, 0.13], *p* = 0.769. Similarly, we found the expected inverse correlation between Intrusion and Determinism scores for Prolificers: *r* = −0.63 [−0.71, −0.55], *p* < 0.001, but not for MTurkers: *r* = −0.04 [−0.16, 0.07], *p* = 0.481 (see Fig. 7). This suggests that MTurkers did not properly understand or interpret these items.^4^

*Explanation for MTurkers’ pattern of answers.* There is still something intriguing about MTurkers’ answers: though their answers often make no sense, they *sometimes* make sense. For example, they massively fail the Intrusion and Time-travel test, but they massively succeed at the Determinism test. Similarly, they massively fail Check B but not Check A. And they give absurd answers to the praise question but seemingly sensible answers to the blame question.

It turns out that this pattern of answer can be easily explained if we suppose that a substantial proportion of MTurkers *answer at random while favoring the right-end of the scale*. Indeed, favoring the right-end of the scale explains why they succeed at Check A (where success involves selecting an answer right of the midpoint) while they fail at Check B (where success involves selecting an answer left of the midpoint). This would also explain why they succeed at the Determinism test (where success involves selecting an answer right of the midpoint) while they fail the Intrusion test (where success involves selecting an answer left of the midpoint). As for the Time-travel test, success involves selecting one particular answer out of 101. Thus, even if this answer is on the rightmost part of the scale, there are plenty of opportunities to get it wrong for someone who would randomly pick answers at random on the right half of the scale.

This interpretation of participants’ answers is also coherent with independent observations. For example, in a series of studies we conducted on Amazon Mechanical Turk in 2020 (Cova & Boudesseul, 2023), we hid two attention checks in a list of items participants were asked to rate their agreement with: “I can shoot lasers with my eyes” and “I am a human being”. Succeeding in the first required clicking on the left-end of a Likert scale, while succeeding in the second required clicking on the right-end of the same Likert scale. Unsurprisingly, in all studies, very few participants failed the second one, but an important proportion failed the first one and casually answered that they could shoot lasers with their eyes. Again, this goes into the idea that an important proportion of MTurkers just rush through surveys while favoring the right side of Likert scales. Moreover, in a longitudinal study documenting the dramatic fall of data quality on Amazon Mechanical Turk, Marshall and colleagues (2023) show that this fall is accompanied by MTurkers providing higher scores on Likert scales, especially when they were expected to give low answers.

Note that data quality has not always been this low on Mechanical Turk and that, at least in our experience, it began to seriously deteriorate somewhere around 2020. Maybe the reader would like to know about the causes of this deterioration, but we actually have no idea and speculation about them is beyond the scope of the present paper.

## Study 5

The main lesson of Studies 1 to 4 was that, whatever the metrics (Intrusion, Determinism or Time-travel), most participants simply seem to *understand* the basic principle of determinism, that is: that, given the past, agents had to act the way they did. The results of Study 4 suggests that previous studies that reported massive failure to understand this basic principle of determinism might have produced unreliable estimates of failure rates due to unreliable samples recruited through Amazon Mechanical Turk. Moreover, the results of Studies 1 to 3 suggest that, compared to other measures of comprehension of determinism, Intrusion measures tended to produce the higher estimates. Thus, it might be that participants’ tendency to fall into Intrusion errors might not be as problematic for experimenters as suggested by previous studies.

However, failing to understand that determinism implies that, given the past, there is only one possible future is not the only type of error participants can fall into. For example, in Study 4, participants’ perception of Epiphenomenalism and Fatalism were very high. Thus, in Study 5, our primary goal was to reduce the rate of these types of errors by using a description of determinism that strives to limit such confusions. This should allow us to reach a better estimate of the minimal proportion of “natural compatibilists” in the target population.

A secondary aim of Study 5 was to compare Nadelhoffer and colleagues’ measures of Epiphenomenalism (which we used in Study 4) with Murray and Nahmias’ measure of Bypassing (which we used in Study 3). Both measures are supposed to capture the same phenomenon, but still behave quite differently. Both Studies 3 and 4 featured a Concrete case, but the success rate for Epiphenomenalism in Study 4 was 18.3%, while the success rate for Bypassing in Study 3 was 65.0%. Moreover, in Study 3, perceptions of Bypassing were sensitive to condition (and were much higher in the Concrete condition, compared to the Abstract one), while perceptions of Epiphenomenalism in Study 4 were mostly insensitive to condition. Our hypothesis is that Bypassing and Epiphenomenalism measures differ in a very important way: while the Bypassing measure exclusively focuses on what *actually* caused the agent’s decision (e.g. “Jeremy's decision to rob the bank had no effect on what he ended up doing”), most items in the Epiphenomenalism measure (except one) focus on *counterfactual* possibilities (e.g. “In Universe A, Jeremy would have decided to rob the bank no matter what he wanted or believed”).^5^ However, past research suggests that what participants consider relevant for free will and moral responsibility is mostly the *actual path*. For example, Andow and Cova (2016) used “fatalistic” vignettes in which an agent’s mental states *actually* caused their decision, but in which their decision would have been the same *no matter what* their mental states would have been. They observed that most participants still ascribed free will and moral responsibility to agents. Thus, it is not clear that (i) Epiphenomenalism is measuring the same thing as Bypassing, and that (ii) Epiphenomenalism is measuring a construct relevant to people’s intuitions about free will and moral responsibility.

### Methods

295 United States residents recruited through Prolific Academic completed our survey. After excluding participants who failed at least one of four attention checks, we were left with 262 participants (136 in the BAD condition, 125 in the GOOD condition; 136 men, 120 women, 6 other; *M*_age_ = 37.00, *SD*_age_ = 13.69).

Participants were presented with a new description of a deterministic Universe (Universe A). The description was divided in two parts. The first part went like this:

*Imagine a universe (let's call it Universe A) in which the laws of nature are so strict they leave no place for chance or randomness. This means that, in Universe A, the same causes will always lead to the same effects. If a machine is in a certain state, there is only one way in which it will possibly act. If an animal is in a certain state, there is only one way in which it will possibly act. And if a human being is in a certain mental state (with certain beliefs, preferences, and values), there is only one way in which they will possibly act.*

*Of course, this does not mean this machine, this animal, or this human being would always act in the same way no matter what. Indeed, if they were in a different state (for example, had different beliefs, desires, or values), they would act differently. But, given their present state, there is only one way in which they will possibly act.*

*Moreover, in Universe A, nothing happens without a prior cause. This means that whatever happens in Universe A happens the way it does because of the way the world was before, together with the laws of nature. Given that these laws leave no room for chance or randomness, this means that, if we brought back Universe A to its exact initial state, the same events would unavoidably unfold. Indeed, the same original cause would inevitably lead to the same results. (Of course, if we brought Universe A back to its original state and modified this state, even slightly, something different would probably occur. But as long as its initial state is the same as it was the first time, the same things will occur.)*

*This also means that if some superintelligent being observed Universe A and knew the laws that govern Universe A, they could use their knowledge of the current state of Universe to compute and deduce everything that will happen in Universe A in the future, including human behaviors.*

Then, on the second page, the presentation continued like this:

*For example, in Universe A, a man named John once decided to have French Fries for lunch. Given his state-of-mind before his decision (including his beliefs, his preferences, and values), it was 100% certain that he would decide to have French Fries. And, if we rebooted Universe A to its initial state, John would always decide to have French Fries on that day, because the same causes will bring the same effects.*

*However, this does not mean that John would have had French Fries French Fries no matter what. Indeed, if he had decided or tried to have something different, he would probably have had something different. And if his beliefs, preferences, or values had been different, he would have made a different decision. Thus, if the past had been different, he would have had different preferences and beliefs, and those different preferences and beliefs would have led him to make a different decision and thus to act differently. But given the past, the laws of his Universe made it so that he had these precise preferences and beliefs, which led him to make this precise decision, and thus to act in this precise way.*

*For example, if he had decided to follow a diet to lose weight, he might have decided not to have French Fries. It is just that, given what happened in the past (his genes, his upbringing, what he did before on that day, etc.), he was in a state-of-mind that made it so that he would necessarily have French Fries.*

Participants were then asked to rate to which extent Universe A is similar to ours (on a 7-point scale from -3 = “Very dissimilar” to 3 = “Very similar”).

After that, participants were presented with a concrete case describing a man named Jeremy Hall, who lives in Universe A and performs a certain action. The action could be either morally BAD (robbing a bank) or morally GOOD (saving a child from drowning). Participants were asked to rate to which extent Jeremy deserved blame and praise for his action, and whether he acted of his own free will.

Participants were then asked to rate their agreement (on a 7-point scale) with 19 statements. Two were attention checks. 12 were inspired by Nadelhoffer and colleagues (in press): 4 Intrusion items, 4 Epiphenomenalism items, and 4 Fatalism items. We also added 3 Bypassing items from Murray and Nahmias (2014). The last two items measured participants’ perception of the concordance between the agent’s action and his Deep Self.

Participants were also asked the following question about time-travel:

*Imagine that, in Universe A, a time-traveler observed Jeremy rob the bank then decided to go back in time 10 minutes before Jeremy made his decision to see whether Jeremy would always make the same decision. Imagine that he did that hundred and hundred of times and never interfered with Jeremy and the events that led to his decision. According to you, which of the following claims is more plausible:*

- *No matter how many times the time-traveler goes back in time, Jeremy will always decide to rob the bank (as long as the time-traveler does not interfere).*
- *If the time-traveler tries long enough, there will be times when Jeremy does not decide to rob the bank (even if the time-traveler does not interfere).*
- *I don't know.*

Finally, participants were asked to fill the same modified Determinism subscale as in Studies 1, 2 and 4. They were also asked to provide information about themselves and to fill a certain number of scales (about their belief in free will, subjective well-being, meaning in life, etc.). However, we won’t be using this information here.

### Results

*Universe*. When asked to rate to which extent our universe was similar to Universe A, participants gave an average answer of −0.11 (*SD* = 1.94). This was not significantly different from the midpoint: *t*(261) = 0.96, *p* = 0.34. 124 participants gave an answer superior to 0, and 120 gave an answer inferior to 0.

*Success rates*. Participants’ success rates for each condition are presented in Table 7. As can be seen, we were successful in diminishing participants’ perceptions of Bypassing/Epiphenomenalism. Most participants also passed the Intrusion/Time travel/Determinism test. However, a majority of participants still failed at the Fatalism measure.

**Table 7:** Mean and standard deviations for participants’ answers to the various questions asked in Study 5, for each condition (BAD vs. GOOD). % indicate success rate (when “Success” is indicated) or the percentage of answers superior to the midpoint (when nothing is indicated). *** in the rightmost column indicate the results of Welch t-tests comparing the BAD and the GOOD conditions (*p < .05, **p < .01, ***p < .001)

	BAD	GOOD
Blame	1.96 (1.59) 83.9%	− 2.46 (1.24)*** 3.2%
Praise	− 2.19 (1.42) 5.8%	2.07 (1.33)*** 87.2%
Free Will	1.04 (2.08) 66.4%	1.21 (2.08) 65.6%
Intrusion	− 0.72 (1.55) Success: 60.6% Failure: 39.4%	− 0.84 (1.59) Success: 64.0% Failure: 36.0%
Time travel	Success: 80.2% Failure: 19.8%	Success: 92.0% Failure: 8.0%
Determinism	1.44 (1.24) Success: 85.4% Failure: 14.6%	1.64 (1.23) Success: 88.8% Failure: 11.2%
Bypassing	− 1.30 (1.60) Success: 84.7% Failure: 15.3%	− 0.57 (1.74)*** Success: 71.2% Failure: 28.8%
Epiphenomenalism	− 0.63 (1.36) Success: 62.0% Failure: 38.0%	− 0.18 (1.60)* Success: 54.4% Failure: 45.6%
Fatalism	− 0.04 (1.48) Success: 46.0% Failure: 54.0%	0.27 (1.47) Success: 37.6% Failure: 62.4%

*Intrusion measures*. As in Studies 1 and 2, Intrusion measures yielded much higher estimates of failure to understand determinism than answers to the Time-travel question: Χ^2^(1) = 36.95, *p* < 0.001, and than Determinism scores: Χ^2^(1) = 41.27, *p* < 0.001.

**Fig. 8:**
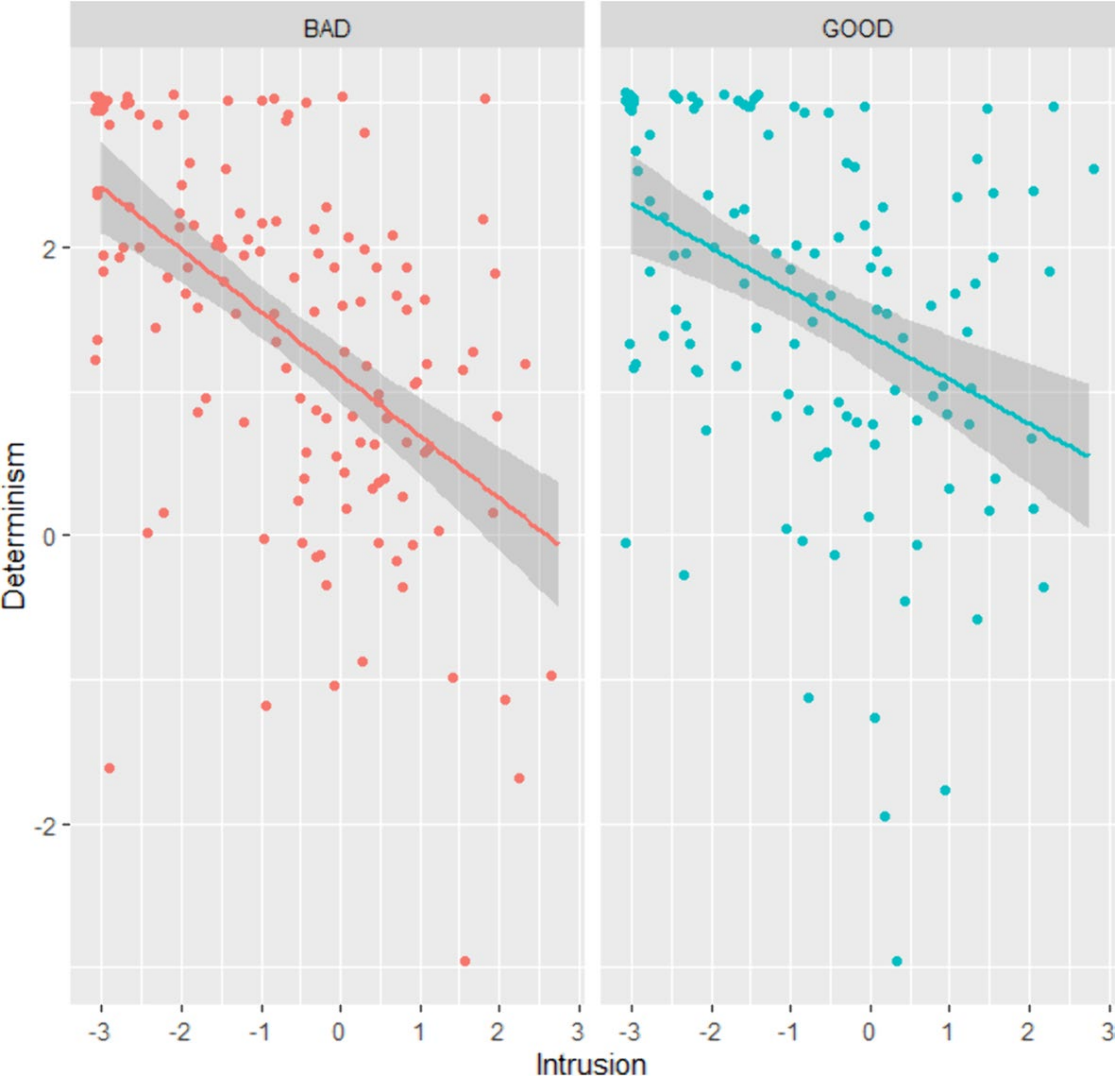
Determinism scores in function of Intrusion scores for each condition and sample (Study 5)

Correlation between Intrusion and Determinism scores (*r* = −0.47, *p* < 0.001) was medium-sized (see Fig. 8). Estimates of success based on Intrusion scores and answers to the Time-travel question agreed on 186 participants out of 262 (71%). This was significantly higher than chance: Χ^2^(1) = 32.24, *p* < 0.001.

*Epiphenomenalism and Bypassing measures*. Bypassing and Epiphenomenalism measures correlated strongly with each other (*r* = 0.75, *p* < 0.001). However, Epiphenomenalism measures yield higher estimates for failure rates compared to Bypassing measures: Χ^2^(1) = 10.31, *p* = 0.001. Thus, depending on which measures one adopts, one will reach different conclusions.

*Fatalism measures*. Despite our efforts, a majority of participants still failed at Fatalism measures. However, there were some issues with these measures. Even though the internal coherence of the Fatalism scale was decent (α = 0.71), the different items yielded different conclusions. Table 8 shows participants’ answers for the different items of this scale. While participants tend to agree with the claim that “there is no sense in which events could have unfolded differently than they did”, that Jeremy “would have ended up acting the way he did no matter what he tried to do”, and that “Jeremy will act the way he did no matter what”, most participants disagreed with the claim that “Jeremy’s action had to happen even if what happened in the past had been different”. This means that, when the question is asked in the most direct way, participants tend to agree with the fact that, had the past been different, Jeremy could have acted otherwise. However, this is *precisely* what the Fatalism measure was supposed to capture and, clearly, 3 items out of 4 seem to fail to capture this very idea.

**Table 8:** Detailed results for each item of the Fatalism measure (Study 5)

Item	*M*	*SD*	% < 0
In Universe A, there is no sense in which events could have unfolded differently than they did	0.38	2.09	37.4%
In Universe A, Jeremy would have ended up robbing the bank/saving the child no matter what he tried to do	0.54	2.10	34.0%
In Universe A, Jeremy will rob the bank/save the child no matter what	0.75	2.20	31.7%
In Universe A, Jeremy’s robbing the bank/saving the child had to happen, even if what happened in the past had been different	− 1.24	1.91	70.2%

*Impact of each type of error of moral responsibility judgment.* To determine to which extent each type of error was important in shaping participants’ intuitions, we ran a multiple regression analysis with moral responsibility judgments (Blame or Praise judgments depending on the case) as dependent variable and Bypassing, Fatalism and Intrusion scores as predictors. Results are presented in Table 9. Taken together, all three errors only explained between 4 and 6% of the variance in moral responsibility judgments, which suggests that these errors did not play a major role in our participants’ judgments.

**Table 9:** Results of multiple regression analysis with Blame/Praise scores as dependent variable and Bypassing, Fatalism, and Intrusion scores as predictors (Study 5). The analysis is performed twice: once for the BAD case, and a second time for the GOOD case. The *r* column indicates zero-order correlations between each factor and Responsibility scores

	*r*	B	β	SE	t	*p*
(a) BAD case						
Intercept	–	–1.92	–	0.17	− 11.20	< .001***
Bypassing	.10	0.18	0.21	0.08	2.23	.028*
Fatalism	− 0.15	− 0.20	− 0.21	0.10	− 2.01	.046*
Intrusion	.13	0.06	0.06	0.09	0.67	.507
*R*^*2*^ = .062						
(b) GOOD case						
Intercept	–	− 2.55	–	0.14	− 18.70	< .001***
Bypassing	.07	0.05	0.07	0.07	0.65	.514
Fatalism	.02	− 0.07	− 0.08	0.09	− 0.74	.459
Intrusion	− .19*	− 0.16	− 0.20	0.07	− 2.09	.038*
*R*^*2*^ = .060* / .040						

*Estimates of compatibilist answers*. For both the Bad and the Good condition, we looked at participants’ answers to the moral responsibility (Blame or Praise) and the free will question after excluding participants who failed at the Intrusion, Time-travel or Determinism test, or the three of them. We also looked at participants’ answers who succeeded at these three tests and also succeeded at the Bypassing measure. We did not take Fatalism measures into account, for the reasons presented earlier (i.e. participants do not seem to interpret Fatalism items in the intended way). Results are presented in Table 10. As can be seen, after excluding participants on all three measures of comprehension of determinism (Intrusion, Determinism, and Time-travel), more than 50% of participants attributed free will, and around 80% attributed moral responsibility (blame or praise).

**Table 10:** Participants’ free will and moral responsibility attributions (blame in the BAD condition, praise in the GOOD condition) after exclusion. Exclusion criteria are presented in the leftmost column. Percentages in the ‘Remaining N’ column indicate the % of remaining participants after exclusion. Percentages in the other columns indicate the % of answers superior to the midpoint (0)

*Exclusion*	*Remaining N*	Blame	Praise	Free will
No exclusion	262	1.96 (1.59) 83.9%	2.07 (1.42) 87.2%	1.12 (2.08) 66.0%
Intrusion	163 (62.2%)	1.72 (1.84) 77.1%	1.93 (1.40) 85.0%	0.66 (2.22) 55.8%
Time travel	225 (85.9%)	1.89 (1.69) 81.8%	2.06 (1.36) 87.0%	0.98 (2.15) 62.7%
Determinism	228 (87.0%)	1.94 (1.63) 83.8%	2.04 (1.37) 86.5%	1.01 (2.11) 64.0%
All 3	145 (55.3%)	1.69 (1.90) 77.5%	1.91 (1.44) 83.8%	0.54 (2.25) 53.1%
Time travel + Bypassing	155 (59.1%)	2.10 (1.46) 86.0%	2.16 (1.30) 88.4%	1.20 (2.06) 67.7%
All 3 + Bypassing	94 (35.9%)	1.96 (1.65) 83.0%	2.07 (1.29) 87.8%	0.77 (2.19) 59.6%

## General Discussion

As we saw, previous research concluded that classic vignettes used by experimental philosophers to investigate folk intuitions about free will and determinism elicited massive comprehension errors. Based on this diagnosis, some have raised the pessimistic possibility that these errors be systematic and unavoidable, meaning that experimenters would not be able to use vignettes to probe folk intuitions about free will and determinism.

Studies 1 to 5 gave us several reasons to think that this initial diagnosis might have been premature. First and foremost, the results of Study 4 suggest that previous studies that aimed at estimating the rate of comprehension errors might have been plagued by unreliable *samples*. Particularly when it comes to *Intrusion* errors, the results of Studies 1 to 4 suggest that most participants do not fall into them and are able to understand that determinism excludes the unconditional ability to do otherwise.

Second, the results of Studies 1 to 5 give us reasons to doubt the validity of the *measures* used to assess the rate of comprehension errors. For example, the results of Study 5 show that the different items composing the Fatalism measure yield very different conclusions, which means that we do not know what these items actually measure and how participants interpret them. Meanwhile, the results of Studies 3 to 5 suggest that the Epiphenomenalism measure used in recent studies behaves very differently from the traditional Bypassing measure and produces much higher failure of comprehension rates. Depending on which measure one uses, the estimate of comprehension errors will thus be different. We find the same problem for Intrusion measures, which tend to yield higher estimates of failure to understand determinism, when compared to the Determinism scale and Time-travel question we used as comparison points. This variability suggests that the tools used to estimate the rate of comprehension errors might not have been the most accurate. For example, looking at Study 5, someone using the Intrusion and Epiphenomenalism measures will reach very different estimates compared to someone using the Time-travel and Bypassing measures (even though they all seem to have face validity and to measure the same constructs at first sight).

Of course, our results do not allow us to conclude that participants in past experiments did not make massive comprehension errors. But they allow us to conclude that we don’t know whether they made massive comprehension errors. To know whether this is the case, new studies with validated measures of comprehension errors will be needed. In the meanwhile, we think that the results of past attempts at estimating the rate of comprehension errors do not justify pessimism about the future, as they are not robust enough to justify pessimism about the past.

Speaking of pessimism, we saw in the introduction that one reason to be pessimistic was the hypothesis that people’s conception of human agency was deeply indeterministic, which meant that participants were bound to fall either for Intrusion or Bypassing errors. The results of Study 1 gave preliminary reasons to doubt this idea: participants were no more likely to fall for Intrusion errors in the case of human agents than in the case of animals or natural events. But, more importantly, in Study 5, we were able to produce a vignette in which an important proportion of participants were able to avoid both Intrusion and Bypassing errors. For example, 61% of participants succeeded both at the Determinism and Bypassing measures, 59% succeeded both at the Time-travel and Bypassing measures, and 41% succeeded both at the Intrusion and Bypassing measures. Depending on which of these three measures you take to be the best measure of Intrusion errors (and, for the reason we just highlighted, we do not think it is the Intrusion measure), this suggests that around half of our participants were able to conceive of determined agents that are neither bypassed, nor endowed with the unconditional ability to do to otherwise. This suggests that the folk conception of agency is not deeply or universally indeterministic.

Of course, one might argue that (around) half of the participants succeeding to understand our vignette is not enough to rejoice. But when exactly is *enough*? Enough should be when the rate is sufficient to reach our goal: getting a good estimate of the rate of “natural compatibilists” and “natural incompatibilists”. So, the question we should ask ourselves is: do the remaining comprehension errors in Study 5 prevent us from reaching an accurate estimate of the rate of “natural compatibilists”? It is to answer this question that we conducted a multiple regression analysis with Bypassing, Fatalism and Intrusion errors as predictors and moral responsibility ratings as our dependent variable. As can be seen in Table 9, these errors explain between 4 and 6% of the total variance (while the same errors explained around 50% of variance in Study 3). Participants’ answers to the Intrusion measure were not significantly related to moral responsibility ratings in the BAD case and were actually *negatively* related to moral responsibility ratings in the GOOD case, meaning that Intrusion errors (or whatever is measured by the Intrusion measure) are not likely to lead us to overestimate the rate of “natural compatibilists”. Accordingly, the results of Table 10 show that estimates of the rate of “natural compatibilists” did not change much after excluding participants who made Bypassing or Intrusion errors.

These results mean that the pessimist must face the following dilemma: either the Bypassing, Fatalism and Intrusion measures accurately captures the corresponding type of errors, but then this means that these errors do not prevent them from drawing conclusions from the results of Study 5; or these errors should prevent them from drawing conclusions from the results of Study 5, but then the measures do not accurately capture them (and we shouldn’t worry about participants’ answers to these measures, as we do not know what they capture).^6^

In any case, the results of Study 5 suggest that pessimism is unwarranted: it seems possible to construct vignettes in which the rate of errors is decreased and in which the impact of these errors on participants’ answers becomes minimal. We do not doubt that other researchers will be able to improve on the vignette we sketched in Study 5. In the meanwhile, our results seem to warrant the very same conclusion that Nahmias and colleagues already drew almost 20 years ago: a majority of people seem to consider that moral responsibility is compatible with determinism.

## Supplementary Information

Below is the link to the electronic supplementary material.

Supplementary file1 (PDF 222 kb)

## Data Availability

All materials and data are publicly available at hosf.io/vr9my/ (DOI: 10.17605/OSF.IO/VR9MY)

## Footnotes

1 For a criticism of the claim that people’s intuitive conception of human agency is fundamentally indeterministic, see the Appendix in Giraud & Cova (in press).

2 Note, however, that answering that there is a chance that the agent might not behave as the computer predicted does not necessarily express a belief in indeterministic agency: it could simply express the belief that the Supercomputer might eventually malfunction (due to a bug or sabotage).

3 In support of this idea, we can point out to the fact that Inamori and colleagues (2024b) ran a similar study using a different recruitment method (Lancers) and found that the average Intrusion score was significantly below the midpoint.

4 Since Nadelhoffer and colleagues (2023) asked separate participants about Intrusion and Fatalism, it is impossible to know whether the same pattern would hold for their participants. Fortunately, Murray and colleagues (forthcoming) have asked both Fatalism and Intrusion questions to the same participants. Depending on the vignettes, correlations between Fatalism and Intrusion range from r = -0.01 to r = -0.42, with an average of r = -0.25, which is quite weak, and suggest that their data might be plagued by similar issues, since their participants were recruited using similar methods through Amazon Mechanical Turk.

5 One way to illustrate the difference between the two measures is through Frankfurt-style cases: some *Epiphenomenalism* statements would apply to such agents, while no *Bypassing* statements would apply.

6 Among the three measures of comprehension of determinism we used in Study 5 (Intrusion, Time-travel and Determinism), the only one that significantly predicts moral responsibility judgments in the intended direction is Determinism in the BAD condition: *r* = -.21, *p* = .016. But this is also the error that participants are less likely to make.
