# Supplementary material for: Failure to Comprehend Determinism or Failure to Measure Comprehension? Methodological Issues in Experimental Philosophy of Free Will
Source: Erkenntnis. 2024 Aug 20;90(7):3215–53. doi: 10.1007/s10670-024-00844-1 (PMC12423183; doi:10.1007/s10670-024-00844-1)
Supplement: Supplementary file 1 — Supplementary file1 (PDF 222 kb) [file 10670_2024_844_MOESM1_ESM.pdf]

Failure to comprehend determinism or failure to measure comprehension?
Methodological issues in experimental philosophy of free will

1. Study 3 - Qualitative analysis of participants' justifications for their answer to the Moral Responsibility question

In Study 3, participants were asked to justify their answer to the moral responsibility question. We decided to analyze these open-ended, qualitative answers to determine to which extent they reflected comprehension errors, such as confusion of determinism with bypassing, and intrusion of indeterministic assumption.

In a first step, both authors (FC and TM) went through participants' justifications and independently created coding categories. They then discussed with each other to agree on a common list of categories. The list can be found in Table S1.

*FREE WILL/FREE : Participants point to the presence (or the absence) of free will OR Participants point to the fact that the agent was (or was not) free, acted (or did not act) freely.<br><br>*CHOICE/CHOOSE: Participants point to the fact that the agent made (or did not make) a choice OR that the agent had or did not have the choice.<br><br>*DECISION: Participants point to the fact that the agent made (or did not make) the decision.<br><br>*OPTIONS/POSSIBILITIES: Participants point to the presence (or absence) of options, or alternative possibilities.<br><br>*UPSTREAM CAUSATION (*IS CAUSED)*: Participants point to the fact that the agent or their decision was (or was not) caused by prior events OR that something prior to the agent's action was caused OR that the agent or their decision was the consequence of what came before.<br><br>*DOWNSTREAM CAUSATION (*HAS CAUSED)*: Participants point to the fact that the agent or their decision *caused* or *determined* the target action.<br><br>*COERCITION/CONSTRAINT/FORCED: Participants point to the fact that the agent was (or was not) coerced, constrained, forced.<br><br>*CONTROL/AGENCY: Participants point to the presence (or absence) of agency or control.<br><br>*INTENTIONAL/ON PURPOSE/PLANNED/PREMEDITATED: Participants point to the fact that the action was (or was not) intentional, done on purpose, planned, premeditated.<br><br>*DETERMINISME/DETERMINE/PREDETERMINE: Participants point to the fact that the action or the universe was (or was not) determined or predetermined.

*FATE: Participants point to the fact that the action was (or was not) fated, pre-organized, or pre-programmed by something greater than the agent.<br><br>*DESIRE/WILL: Participants point to the fact that the agent performed (or did not perform) an action in line with their desires, what they wanted, or what they willed.<br><br>*MORAL JUDGMENT: Participants point to the fact that the agent's action was good/bad, or right/wrong, praiseworthy/blameworthy, just/unjust.<br><br>*OUTLIER: Participants give an irrelevant answer, or a justification that goes against their initial answer.

Table S1. List of categories used for the qualitative analysis of participants' justifications (Study 3).

Then, in a second step, the second author (TM) went through all justifications and, for each justification and each category, rated (i) whether the category was mentioned in the justification, and (ii) if it was mentioned, whether the relevant concept was mentioned as being present (e.g. presence of FATE) or absent (e.g. absence of FATE). Results of this coding procedure can be found in Tables S2 and S3.

Universe A				
	Abstract case		*Concrete case*	
	Answer <= 0	Answer > 0	Answer <= 0	Answer > 0
Free will (YES)	0.0%	3.8%	1.6%	1.6%
Free will (NO)	19.0%	5.7%	11.5%	4.0%
Choice (YES)	2.9%	20.8%	1.6%	11.2%
Choice (NO)	24.1%	5.7%	36.1%	4.0%
Decision (YES)	16.8%	15.1%	14.8%	28.8%
Decision (NO)	8.0%	0.0%	4.9%	0.8%
Options (YES)	0.0%	0.0%	0.0%	0.0%
Options (NO)	1.5%	0.0%	1.6%	0.8%
Upstream causation (YES)	19.7%	15.1%	11.5%	4.8%
Upstream causation (NO)	0.0%	0.0%	0.0%	0.0%
Downstream	2.2%	11.3%	3.3%	6.4%

causation (YES)				
Downstream causation (NO)	0.0%	0.0%	0.0%	0.0%
Constraint (YES)	1.5%	0.0%	1.6%	0.8%
Constraint (NO)	0.0%	0.0%	0.0%	0.8%
Agency (YES)	0.7%	5.7%	0.0%	1.6%
Agency (NO)	19.0%	0.0%	4.9%	0.0%
Intention (YES)	0.7%	1.9%	0.0%	16.0%
Intention (NO)	0.7%	0.0%	1.6%	0.0%
Determinism (YES)	25.5%	11.3%	26.2%	5.6%
Determinism (NO)	0.0%	0.0%	1.6%	1.6%
Fatalism (YES)	10.2%	1.9%	6.6%	3.2%
Fatalism (NO)	0.0%	1.9%	0.0%	0.8%
Desires (YES)	0.0%	3.8%	0.0%	5.6%
Desires (NO)	0.7%	0.0%	1.6%	0.0%
Morality (YES)	3.6%	15.1%	3.3%	19.2%
Morality (NO)	15.3%	3.8%	11.5%	0.8%
Special cases	3.6%	20.8%	16.4%	8.0%

Table S2. % of answers falling into each category for the Deterministic Universe (Universe A) in function of condition (Abstract vs. Concrete) and of participant's answer to the Responsibility question (above the midpoint or not).

Universe B				
	Abstract case		*Concrete case*	
	Answer <= 0	Answer > 0	Answer <= 0	Answer > 0
Free will (YES)	0.0%	27.1%	0.0%	5.9%
Free will (NO)	0.0%	0.6%	0.0%	0.0%
Choice (YES)	10.0%	43.6%	0.0%	23.4%

Choice (NO)	0.0%	0.0%	0.0%	0.0%
Decision (YES)	50.0%	43.6%	22.2%	43.1%
Decision (NO)	0.0%	0.0%	0.0%	0.0%
Options (YES)	0.0%	5.5%	0.0%	5.9%
Options (NO)	0.0%	0.0%	0.0%	0.5%
Upstream causation (YES)	20.0%	2.2%	0.0%	0.0%
Upstream causation (NO)	0.0%	2.8%	0.0%	2.1%
Downstream causation (YES)	0.0%	5.5%	0.0%	7.4%
Downstream causation (NO)	0.0%	1.1%	0.0%	0.0%
Constraint (YES)	0.0%	1.1%	0.0%	0.5%
Constraint (NO)	0.0%	1.7%	0.0%	2.1%
Agency (YES)	0.0%	3.9%	11.1%	1.6%
Agency (NO)	0.0%	0.6%	0.0%	0.5%
Intention (YES)	0.0%	0.0%	0.0%	26.6%
Intention (NO)	0.0%	0.6%	0.0%	0.0%
Determinism (YES)	0.0%	1.7%	0.0%	0.0%
Determinism (NO)	0.0%	3.9%	0.0%	2.7%
Fatalism (YES)	10.0%	1.1%	0.0%	0.5%
Fatalism (NO)	0.0%	4.4%	0.0%	0.0%
Desires (YES)	0.0%	3.3%	0.0%	3.7%
Desires (NO)	0.0%	0.0%	0.0%	0.0%
Morality (YES)	0.0%	23.2%	22.2%	15.4%
Morality (NO)	0.0%	0.0%	11.1%	0.5%
Special cases	50.0%	5.0%	77.8%	0.5%

Table S3. % of answers falling into each category for the Indeterministic Universe (Universe B) in function of condition (Abstract vs. Concrete) and of participant's answer to the Responsibility question (above the midpoint or not).

In Tables S2 and S3, the "special cases" category regroup either (i) justifications that were too unclear to be coded, or (ii) the content of which contradicts the numerical answer participants gave to the other questions (for example, a participant gave a positive answer to the Free Will and Responsibility questions but then declared "all I can do is reiterate my belief that we don't have free will in the sense that many people believe").

The results presented in Table S2 seem to show little sign of intrusion. Participants who denied moral responsibility appealed to the presence of "upstream causation" (19.7% for the Abstract case, 11.5% for the Concrete case), but no participant who attributed moral responsibility actually denied the presence of upstream causation (0% in both cases). Participants who denied moral responsibility also appealed to "determinism" (25.5% for the Abstract case, 26.2% for the Concrete case), but very few participants who attributed moral responsibility actually denied the presence of determinism (0% in the Abstract case, 1.6% in the Concrete case).

There was some confusion between determinism and fatalism: 10.2% of participants who denied moral responsibility in the Abstract case and 6.6% of participants who denied moral responsibility in the Concrete case appealed to fate-related concepts in their justifications.

Denials of moral responsibility were mainly motivated by appeal to the absence of choice (24.1% in the Abstract case and 36.1% in the Concrete case). Some might see this as a sign of confusion between Determinism and Bypassing, but it might be reasonably argued that Determinism *does* preclude a certain type of choice (unconditional choice) and that these justifications do not necessarily reflect comprehension errors.

Attributions of moral responsibility were mainly motivated by appeal to the presence of a decision. There is no reason to think that this reflects a comprehension error on behalf of participants.

Overall, our analysis detected very few rejections of determinism from our participants. Of course, a participant may reject the vignette's deterministic assumptions without mentioning this rejection in their justification. But this gives us additional reasons to think that participants are not massively misinterpreting the vignettes.

2. Study 4 - Results for the morally good case

In Study 4, one third of participants recruited through Prolific Academic were assigned to a *Morally Good* condition, the results of which are not presented in the main manuscript. The results for this condition are presented in Table S4, alongside the results of the *Morally Bad* condition (for Prolificers only).

	Bad	*Good*
Blame	1.08 (2.25)<br>64.2%	-2.16 (1.53)<br>07.0%
Praise	-2.39 (1.22)<br>2.5%	1.86 (1.67)<br>78.9%

Free Will	0.24 (2.40)<br>48.3%	0.52 (2.28)<br>50.0%
Epiphenomenalism	1.14 (1.53)<br>Success: 18.3%	1.19 (1.42)<br>Success: 16.7%
Fatalism	1.32 (1.34)<br>Success: 14.2%	1.28 (1.27)<br>Success: 8.8%
Intrusion	-1.27 (1.67)<br>Success: 73.3%	-1.48 (1.58)<br>Success: 76.3%
Time-travel	89.3 (24.91)<br>Success: 75.0%	92.2 (19.58)<br>Success: 75.4%
Determinism	1.85 (1.22)<br>Success: 91.7%	2.01 (0.95)<br>Success: 94.7%
Check A	2.50 (1.33)<br>Success: 91.7%	1.90 (1.90)<br>Success: 78.1%
Check B	-2.43 (1.43)<br>Success: 90.8%	-1.68 (2.15)<br>Success: 80.7%
N	120	114

Table S5. Mean and standard deviations for participants' answers to the various questions asked in Study 4, for the *Bad* and *Good* concrete cases (Prolificers only). % indicate success rate (when "Success" is indicated) or the percentage of answers superior to the midpoint (when nothing is indicated).
